# Anti-Mesothelin CAR-NK cells as a novel targeted therapy against cervical cancer

**DOI:** 10.3389/fimmu.2024.1485461

**Published:** 2024-12-16

**Authors:** Ivana Kutle, Robert Polten, Jan Lennart Stalp, Jens Hachenberg, Felix Todzey, Ralf Hass, Katharina Zimmermann, Juliane von der Ohe, Constantin von Kaisenberg, Lavinia Neubert, Jan C. Kamp, Dirk Schaudien, Ann-Kathrin Seyda, Peter Hillemanns, Rüdiger Klapdor, Michael Alexander Morgan, Axel Schambach

**Affiliations:** ^1^ Institute of Experimental Hematology, Hannover Medical School, Hannover, Germany; ^2^ Department of Gynecology and Obstetrics, Hannover Medical School, Hannover, Germany; ^3^ Institute of Pathology, Hannover Medical School, Hannover, Germany; ^4^ Biomedical Research in Endstage and Obstructive Lung Disease Hannover (BREATH), German Center for Lung Research (DZL), Hannover, Germany; ^5^ Department of Respiratory Medicine and Infectious Diseases, Hannover Medical School, Hannover, Germany; ^6^ Fraunhofer Institute for Toxicology and Experimental Medicine, ITEM, Hannover, Germany; ^7^ Department of Gynecology and Obstetrics, Albertinen Hospital Hamburg, Hamburg, Germany; ^8^ Division of Hematology/Oncology, Boston Children’s Hospital, Harvard Medical School, Boston, MA, United States

**Keywords:** cervical cancer, immunotherapy, chimeric antigen receptor (CAR), CAR-NK cells, Mesothelin, chemotherapy, CAR-T cells

## Abstract

Resistance to the currently available treatment paradigms is one of the main factors that contributes to poor outcomes in patients with advanced cervical cancer. Novel targeted therapy approaches might enhance the patient’s treatment outcome and are urgently needed for this malignancy. While chimeric-antigen receptor (CAR)-based adoptive immunotherapy displays a promising treatment strategy for liquid cancers, their use against cervical cancer is largely unexplored. This study used alpharetroviral SIN vectors to equip natural killer (NK) cells with a third-generation CAR (including CD28 and 4-1BB co-stimulatory domains) targeting Mesothelin, which was identified to be highly expressed on primary human cervical cancer tissues and cervical cancer cell lines in this and other studies. Anti-Mesothelin CAR-NK cells demonstrated high cytotoxicity against cervical cancer cells in 2D and 3D culture models, which corresponded to increased degranulation of CAR-NK-92 cells upon exposure to Mesothelin^+^ target cells. Mesothelin^-^ cervical cancer cells were generated by CRISPR-Cas9-mediated knockout and used to show target antigen specificity of anti-Mesothelin CAR-NK-92 cells and primary NK cells derived from different healthy donors in co-culture experiments. Combination of anti-Mesothelin CAR-NK-92 cells with chemotherapy revealed increased elimination of cancer cells as compared to monotherapy settings. Our findings indicate the promise of anti-Mesothelin CAR-NK cells as a potential treatment option against cervical cancer, as well as other Mesothelin^+^ malignancies.

## 1 Introduction

Targeted therapy approaches are increasingly implemented in cancer therapy regimens, often complementing conventional treatments (*e.g.*, chemotherapy, radiation). Among these novel strategies, targeted cell therapy approaches harnessing the body’s own immune system have emerged prominently. Particularly, T cells modified to express chimeric antigen receptors (CAR-T) that re-direct the immune cells towards a specific tumor-associated antigen have demonstrated promising results, especially against B cell lymphomas (1, 2). The success of CAR-based therapies culminated in the FDA-approval of seven CAR-T cell therapies to date for use to treat liquid cancers. However, challenges persist, especially in the context of solid tumors, which necessitate exploration of alternative cell types and antigens (3). Natural killer (NK) cells have garnered attention for CAR-based therapy due to their inherent anti-cancer properties, minimal induction of cytokine release syndrome (CRS), and lack of MHC restrictions, making them ideal candidates for off-the-shelf cell therapy (4).

Cervical cancer is a solid malignancy that is a significant health threat to women worldwide, with approximately six hundred thousand incidences and over three hundred thousand deaths yearly. While early cervical cancer is usually successfully treated by radical surgery or chemoradiation, there are only limited treatment options for recurrent or metastatic cervical cancer. Only a few studies are investigating the use of CAR-T cells against cervical cancers, with most of them being ongoing (5). Here, Mesothelin is a promising potential target antigen for CAR therapies, as it is highly expressed on a multitude of different cancers, including the majority of cervical cancers, but absent in most healthy adult tissues, with limited expression reported in mesothelial linings of the peritoneum, pleura, and pericardium (6, 7). Mesothelin is a 40-kDa glycosylphosphatidylinositol (GPI)-anchored cell surface protein that is localized to the cell surface after soluble 31-kDa megakaryocyte potentiating factor (MPF) is cleaved from a Mesothelin-precursor (8). A study performed Mesothelin knockout experiments in mice and identified no anatomical, growth, or reproduction abnormalities, leaving the functional role of Mesothelin widely unknown (9). However, its binding capability to MUC16 indicates potential roles in cancer cell adhesion and metastasis (10). Some studies already assessed the feasibility of Mesothelin CAR-T cells, *e.g.*, against pancreatic and ovarian cancer, and showed strong *in vitro* and *in vivo* anti-cancer effects (11, 12). Anti-Mesothelin CAR-T cells resulted in responses or stable disease in clinical trials of patients with gynecologic malignancies. Only limited side-effects were observed, highlighting the potential of targeted immune cell-based treatment strategies (13, 14). However, as these studies rarely included cervical cancer as targets, the efficacy of CAR-based strategies remains to be further characterized in this disease (15).

In the present study, we investigated the potential of a novel CAR-NK cell-based therapy against cervical cancer. Here, we used alpharetroviral SIN vectors to deliver a third-generation anti-Mesothelin CAR into NK-92 cells and primary human NK cells. Anti-Mesothelin CAR-NK anti-cervical cancer activity was assessed in 2D and 3D co-culture experiments that showed more effective elimination of cancer cells by CAR-NK cells as compared to control NK cells. These findings highlight the potential of CAR-NK cells as a promising therapeutic strategy against cervical cancer.

## 2 Material and methods

### 2.1 Cells and cell culture

HEK-293T cells (DSMZ, Brunswick, Germany) were cultivated in DMEM medium (Thermo Fisher Scientific, Waltham, MA, USA) supplemented with 10% fetal bovine serum (FBS Standard/Good, PAN-Biotec, Aidenbach, Germany) and 1% penicillin/streptomycin (P/S; PAN-Biotec). Cervical cancer cell lines, CaSki, SiHa and Hela (ATCC, Manassas, VA, USA) were cultivated in RPMI 1640 medium (PAN-Biotec) supplemented with 10% FBS, 1% P/S, 1 mM sodium pyruvate (PAN-Biotec). NK-92 cells (DSMZ) (16) were cultivated in RPMI 1640 medium (PAN-Biotec) supplemented with 10% FBS, 1% P/S, 1 mM sodium pyruvate (PAN-Biotec) and 400 U/mL human IL2 (PeproTech, Winterhude, Germany). This investigation was approved by the Hannover Medical School ethics committee (Nr. 10948_BO_K_2023; Nr. 6090_BO_K_2018), and cervical cancer patient samples as well as healthy donor samples were collected upon informed consent in accordance with the principles of the Declaration of Helsinki. Primary NK cells were isolated from cord blood- and peripheral blood-derived mononuclear cells (referred as pCB -NK and pPB -NK cells) with the human NK Cell Isolation Kit (Miltenyi Biotec, Bergisch Gladbach, Germany) according to the manufacturer’s protocol. In brief, the cells were labeled with NK Cell Biotin-Antibody Cocktail and, after incubation and washing, with NK Cell MicroBead Cocktail prior to enrichment on columns via a MACS Separator. The collected flow-through consisted of enriched unlabeled CD56^+^ NK cells. The purity of isolated cells was quantified via flow cytometric staining for CD56 and CD3. Isolated CD56^+^ CD3^-^ NK cells were cultivated in complete NK MACS Medium (Miltenyi Biotec) supplemented with 5% human serum (c.c.pro, Oberdorla, Germany), 1% P/S (PAN-Biotech), 1% NK MACS supplement (Miltenyi Biotec), 500 IU/mL of hIL-2 (Proleukin^®^, Novartis), and 70 ng/mL hIL-15 (PeproTech, Hamburg, Germany). Primary T cells were isolated from peripheral blood-derived mononuclear cells (PBMCs) (referred as pPB -T cells) using the Pan T cell Isolation Kit (Miltenyi Biotec) according to the manufacturer’s protocol. The isolated cells were stained for CD4 and CD8 and analyzed by flow cytometry to quantify CD4^+^ and CD8^+^ T cell populations. The isolated T cells were cultivated in complete TexMACS medium (Miltenyi Biotec) supplemented with 3% human serum (c.c.pro, Oberdorla, Germany), 1% P/S (PAN-Biotech), 500 IU/mL of hIL-7, 84 IU/mL of hIL-15 (Miltenyi Biotec). All cells were cultured under standard humidified conditions at 37°C and 5% CO_2_.

### 2.2 Antigen and transgene detection

Flow cytometry was used for the evaluation of surface markers or transgene expression. The following antibodies were used: anti-Mesothelin-APC (REA1057, Miltenyi Biotec), anti-CD3-APC (OKT3, BioLegend, San Diego, CA, USA), anti-CD56-FITC (REA196, Miltenyi Biotec), anti-CD4-FITC (M-T466, Miltenyi Biotec), anti-CD8- PE-Cy7 (SK1, Biolegend). To increase the specificity of the stainings, cells were incubated with an FcR blocking reagent (130-059-901, Miltenyi Biotec) for 10 minutes at 4°C. For staining of the degranulation marker CD107a, co-cultures were incubated with anti-CD107a-APC (Miltenyi Biotec) for 1 h at 37°C and 5% CO_2_ with shaking. Afterwards, Brefeldin A (1:1,000; BioLegend) and Monensin (1:1,000; BioLegend) were added for an additional incubation for 4 hours at 37°C and 5% CO_2_ with shaking. Stained cells were measured via CytoFLEX S (Beckman Coulter, Brea, CA, USA) and analyzed via FlowJo software (Tree Star Inc., Ashland, OR, USA). To investigate the antigen expression after cisplatin treatment, cells were pre-treated with the IC50 value calculated for the respective cell line for 72 hours before the measurement by flow cytometry.

### 2.3 Immunohistochemistry stainings

Immunohistochemistry (IHC) stainings were performed on paraffin sections. To remove the paraffin, sections were treated with xylene (2x 10 minutes) and then rehydrated using descending concentrations of ethanol (100%, 90%, 70%, 50%, H_2_0) for 2 minutes each. To perform antigen retrieval, sections were pre-treated with Tris (T-EDTA buffer #ZUC029, Zytomed Systems) (pH9) or citrate (citrate buffer #ZUC028) (pH6) for 30 minutes at 98°C, followed by 10 minutes of H_2_0. Next, slices were incubated for 5 minutes with H_2_O_2_, washed (washing buffer #ZUC020, Zytomed Systems) (5 minutes), blocked (blocking solution #ZUC007-100, Zytomed Systems) (5 minutes), washed (5 minutes), and stained for one hour with the respective antibody (antibody diluent #ZUC025, Zytomed Systems). Afterwards, slices were washed (5 minutes) and then incubated for 30 minutes with a horseradish peroxidase (HRP) polymer (#ZUC050/ZUC032, Zytomed Systems). After washing (5 minutes), the slices were incubated for 15 minutes with 3,3’-diaminobenzidine (DAB) solution (DAB substrate kit #DAB057, Zytomed Systems). After a 5-minute incubation step in H_2_0, a hematoxylin counterstain was performed. Thereafter, the stained slices were digitalized with the Nano Zoomer S210 (Hamamatsu Photonics, Shizuoka, Japan). For analysis, the Visiopharm (Visiopharm, Hørsholm, Denmark) software with the nuclei detection AI (brightfield) application was used to count the DAB-positive and negative cells. The following antibodies were used: anti-Mesothelin (D9R5G, Cell Signaling Technology), anti-CD3 (2GV6, Roche Diagnostics, Mannheim, Germany), anti-CD11b (EPR1344, abcam), anti-CD19 (CD19-163-L-CE, Leica, Wetzlar, Germany), anti-CD31 (JC70, Roche Diagnostics), anti-CD45 (2B11 & PD7/26, Roche Diagnostics), anti-CD56 (MRQ-42, Roche Diagnostics).

### 2.4 Cloning of vectors and viral vector production

The sequence of the tumor-specific anti-Mesothelin scFv HN1 (17) was codon-optimized for human codon usage; cryptic splice sites were removed, and the GC content was improved to optimize transcriptional processing and protein expression. The sequence was synthesized by GeneArt (Thermo Fisher Scientific, Regensburg, Germany). The scFv was cloned into a third-generation CAR containing CD28, CD137 (4-1BB) and CD3ζ domains. Third-generation CARs carrying CD28, 4-1BB and CD3ζ domains were cloned into self-inactivating (SIN) alpharetroviral vector plasmids carrying an IRES-eGFP cassette for easy detection described previously (18). Specific primers were used to introduce the FLAG-tag on the N-terminus of scFv to enable its detection on the surface of modified NK cells.

### 2.5 Production of retroviral vector supernatants

Transfection was performed in HEK-293T cells, which were seeded the day before at a density of 5 × 10^6^ per 10 cm dish. Alpharetroviral vectors were pseudotyped with RD114TR, while lentiviral vectors were pseudotyped with VSVg. All viral vector supernatants were generated using HEK-293T cells and calcium-phosphate transfection in combination with 25 µM chloroquine. Split-packaging design (19) used for alpharetroviral vector production was 5 μg alpharetroviral vector construct, 2.5 μg pcDNA3-α-gag/pol.co (19) and 2 μg phCMV-RD114TR (20) and for lentiviral vector (LV.SFFV.mCherry.pre) production, 5 μg lentiviral vector construct, 12 μg pcDNA3.GP.4xCTE, 6 μg pRSV-Rev and 2 μg pMD.G-VSVg (21). Viral supernatants were harvested 24 and 48 hours after transfection, filtered through Millex-GP 0.22 μm filters (Millipore, Schwalbach, Germany), concentrated via ultracentrifugation at 4°C at 12,259 ×g for 16 hours (RD114/TR pseudotyped particles) and 76,615 ×g 2 hours (VSVg pseudotyped particles), and stored at −80°C.

### 2.6 Transduction of cell lines with retroviral vectors

Transduction of NK cells with alpharetroviral vectors was done on Retronectin (Takara Bio, Otsu, Japan) coated 48-well plates. Wells were loaded with viral supernatant and centrifuged at 400 ×g and 4°C for 30 minutes. Next, 1 × 10^5^ NK cells were seeded per well. For transduction of primary NK cells, viral vector supernatants were applied at MOI 30 and transduction was repeated twice on consecutive days. Here, we supplemented the medium with 1 mg/mL Synperonic F-108 (Sigma-Aldrich, Missouri, United States) to enhance the transduction efficacy of primary NK cells. For transduction of primary T cells, 1 x 10^6^ T cells were seeded in a 48-well plate and activated using TransAct (1:100, Miltenyi Biotec) for 24 hours. The next day, viral vector supernatants were applied at MOI 20 in addition to polybrene (4 µg/mL, Sigma Aldrich, Missouri, USA) for 48 hours. Subsequently, the T cells were centrifuged for 10 minutes at 300 ×g and resuspended in complete TexMACS medium. Four days after the T cell transduction, the medium was replaced with TexMACS medium without human serum and cells were further cultivated and expanded for 7 days before performing functional experiments. For transduction with the lentiviral vector LV.SFFV.mCherry.pre encoding the mCherry gene, 1 × 10^5^ SiHa or HeLa cells were seeded on a 24-well plate. Next, viral supernatant was added to culture medium containing protamine sulfate (4 µg/mL). Viral supernatants were titrated on the NK-92 cells for alpharetroviral vector supernatants and on HT1080 cells for mCherry lentiviral vector supernatants at least 7 days post-transduction. Transduction efficiency was calculated based on the percentage of FLAG-expressing or mCherry-expressing cells quantified by flow cytometry. Transduced NK cells and target cancer cells were sorted at the cell sorting core facility at Hannover Medical School.

### 2.7 Vector copy number determination

Genomic DNA (gDNA) was isolated from unmodified and genetically modified NK-92 and primary NK cells with the QIAamp DNA Blood Mini Kit (Qiagen N.V., Venlo, Netherlands) according to the manufacturer’s instructions. For mean vector copy number (mVCN) determination, gDNA was analyzed by real-time quantitative polymerase chain reaction (qPCR) with TaqMan probe by using TaqMan Fast Advanced Master Mix (Applied Biosystems-ABI, Life Technologies, Thermo Fisher Scientific, Waltham, MA, USA). The primers were specific for the woodchuck hepatitis virus posttranscriptional regulatory element (*WPRE*), i.e. *WPRE* forward GAGGAGTTGTGGCCCGTTGT; *WPRE* reverse TGACAGGTGGTGGCAATGCC; *WPRE* probe CTGTGTTTGCTGACGCAAC; and the genomic polypyrimidine tract binding protein 2 (*PTPB2*), i.e. *PTBP2* forward TCTCCATTCCCTATGTTCATGC; *PTBP2* reverse GTTCCCGCAGAATGGTGAGGTG; *PTBP2* probe ATGTTCCTCGGACCAACTTG, as previously described (22). The qPCR was accomplished on a StepOnePlus device (Applied Biosystems).

### 2.8 Immunoblotting

Cell pellets of modified and unmodified control NK-92 cells were lysed in lysis buffer (50 mM Tris-HCl, pH 7.5, 150 mM NaCl, 100 mM NaF, 1% Triton X-100), supplemented with freshly added protease inhibitor (1x cOmplete™, Mini Protease Inhibitor Cocktail, Roche Diagnostics), 1 mM Na_3_VO_4_ and 1 mM DTT. Cell lysates were incubated on ice for 20 minutes and centrifuged for 15 minutes at high speed and 4°C. Total protein concentrations of cell lysates were determined with the Coomassie dye-binding assay (Bio-Rad Laboratories, Hercules, CA, USA). Proteins (25 µg of whole cell lysates per sample) were separated by SDS-PAGE and transferred to nitrocellulose membranes. Membranes were blocked with 5% milk in Tris-buffered saline containing 0.1% Tween 20 (TBST), followed by incubation with HRP-coupled anti-CD3ζ antibody (1:1,000, Santa Cruz Biotechnology, CA, USA) in 5% milk in TBST. After detection, membranes were stripped and reprobed with anti-GAPDH-HRP (1:10,000, GeneTex/BIOZOL, Eching, Germany) in 5% milk in TBST. Signals were visualized using the Super Signal West Femto maximum sensitivity substrate (Thermo Fisher Scientific) and FusionFX (PeqLab) imaging system.

### 2.9 Fluoroskan Ascent™ FL

Cervical cancer SiHa cells stably expressing mCherry fluorescent protein were seeded in flat bottom 96-well plates (Sarstedt, Nümbrecht, Germany) at a density of 8,000 cells/well. NK-92 cells were added at the designated effector/target (E:T) ratios the next day. At several time points, fluorescence was measured using the microplate fluorometer Fluoroskan Ascent™ FL (Thermo Fisher Scientific, Waltham, MA, USA). To remove NK-92 suspension cells from the wells, the culture medium was completely removed by inverting the plates and blotting them against clean paper towels before acquiring fluorescence measurements. Two hundred microliters 5% (w/v) SDS was added into each well, followed by the detection of the fluorescence intensities of mCherry in the cell homogenate with excitation at 584 nm and emission at 612 nm (23, 24).

### 2.10 Flow cytometry-based cytotoxicity assay

Cervical cancer cell lines (SiHa, HeLa) cells stably expressing mCherry were seeded in adherent 48-well plates (Sarstedt, Nümbrecht, Germany) at a density of 11,000 cells/well using 400 µL RPMI medium. The next day, effector NK cells (unmodified, extracellular truncated CAR (=ΔCAR), anti-Mesothelin CAR) were added to the pre-seeded target cells in the respective E:T ratios in 400 µL complete RPMI (incl. a final conc. of 400 IU/mL hIL-2). At different time points (0h, 24h, 48h), cells were harvested (adherent cells were trypsinized), washed with 500 µL PBS, and subsequently measured in 100 µL FACS buffer (PBS containing 2% FBS and 0.4% EDTA) with a Cytoflex S flow cytometer (Beckman Coulter). For analysis, the percentage of remaining target cells (identified by mCherry-positivity) was calculated and normalized to the 0 hour time point, to identify the fold change as a readout for cytotoxicity.

### 2.11 Live-cell imaging-based cytotoxicity assay

For live-cell imaging assays, 8,000 cells/well cervical cancer cells (SiHa mCherry/HeLa mCherry) were seeded onto 96-well adherent plates (Sarstedt, Nümbrecht, Germany) in 100 µL of complete RPMI. Effector NK cells (unmodified, ΔCAR, anti-Mesothelin CAR) were added the next day in the respective E:T ratios in 100 µL of complete RPMI, supplemented with a final concentration of 400 IU/mL hIL-2. Afterwards, the cells were measured every two hours for a total duration of three days using an automated live-cell imaging device, CELLCYTE X™ (Discover Echo, San Diego, CA, USA), or Incucyte^®^ SX5 (Sartorius, Göttingen, Germany). For the combinatorial approach with chemotherapy, cervical cancer cells were pre-treated with the respective IC50 concentration of cisplatin for a total duration of 72 hours. Afterwards, the cisplatin-containing medium was removed, and the cells were seeded in 96-well adherent plates as described above.

### 2.12 CRISPR-Cas9 knockout of Mesothelin and validation

To knock out Mesothelin in SiHa cells via CRISPR-Cas9, nucleofection of the RNP complexes (Cas9 protein, crRNA and tracrRNA, Integrated DNA Technologies, Coralville, IA, USA) was performed using the Lonza 4-D-Nucleofector according to the manufacturer’s protocol CN-114. To prepare the ATTO-labelled guide RNAs, crRNA and tracrRNA oligos targeting different loci on the *MSLN* gene were mixed in equimolar concentrations with a final concentration of 100 µM.

sgRNA target site #1: CCGAAGTGCTGCTACCCCGG
sgRNA target site #2: CTGTTGGGGTCCTGTGGGAC

Afterwards, the mix was incubated at 95°C for 5 minutes using a thermal cycler, kept at room temperature (RT) for 10 minutes, and then placed on ice until further usage. To prepare RNP complexes, crRNA:tracrRNA duplexes and Cas9 nuclease V3 were mixed and incubated at room temperature for 15 minutes. SiHa cells (2 x 10^5^) were washed twice with PBS at RT and resuspended in 20 µL SE Cell Line 4D-Nucleofector™ X Solution. The next day, nucleofection efficiency was measured by analyzing ATTO-labelled cells via flow cytometry. The Mesothelin^-^ population of SiHa cells were purified by fluorescence-activated cell sorting. To evaluate the knockout on a DNA level, we performed an amplification of sgRNA target sites with the Platinum™ Hot Start PCR Master Mix (2x) (Cat.#13000012) according to the manufacturer’s protocol. PCR amplicons were purified by agarose gel electrophoresis, and DNA bands were excised and eluted via column purification. Isolated DNA was sequenced using the following Primers:

Primer for sgRNA target site #1: AAGTGTCCTCACGCTGCTGATA
Primer for sgRNA target site #2: GGTACCACAGAAGTTTGCTCTG

To evaluate the knockout on a protein level, we performed flow cytometric experiments to determine the Mesothelin surface expression. Mesothelin knockout on the genomic level was assessed by amplification of the target sequences by PCR, sequencing the amplicons, and calculation of the knockout efficiency using the TIDE program.

### 2.13 Spheroid culture

SiHa spheroids were generated in ultra-low attachment (ULA) 96-well round bottom plates (S-Bio, Hudson, NH, USA). 1,000 SiHa cells were seeded in 100 µL complete RPMI medium per well. The plates were centrifuged at 125 xg for 10 min and incubated for 3-5 days in a humidified incubator at 37°C with 5% CO_2_. In experiments with Mesothelin knockout cells, 500 Mesothelin knockout cells and 500 SiHa Mesothelin^+^ cells were mixed and seeded for spheroid formation as described above. After successful formation, compact spheroids were further applied in the 3D cytotoxicity assays.

### 2.14 Cytotoxicity assays on 3D spheroid model

The CAR-NK cells and ratios that showed the most promising results in cytotoxic assays from 2D settings were further tested on the spheroid model. NK cells adjusted to contain the appropriate number of cells in 100 µL complete RPMI containing hIL-2 in a concentration of 800 IU/mL were added to the formed spheroids. The final medium volume per well was 200 µL with 400 IU/mL human IL-2. The wells were imaged using a fluorescence automated imaging analyzers CELLCYTE X™ (Discover Echo, San Diego, CA, USA), or Incucyte^®^ SX5 (Sartorius, Göttingen, Germany) immediately after adding NK cells up to 4 days of co-culture with 4 hours intervals. For cytotoxicity assays using primary CAR-NK cells, primary CAR-NK cells were added in 100 µL complete NK MACS medium in the respective cell ratios to the formed spheroids. Cytotoxicity assays on 3D spheroids and primary CAR-T cells were performed in 100 µL complete TexMACS medium without human serum and 100 µL RPMI medium.

### 2.15 Statistical analysis

Statistical analysis was performed using GraphPad Prism 5 or 9 software (GraphPad Software, San Diego, CA, USA) and two-way ANOVA test. Error bars in all figures represent the standard deviation of the mean. Statistical significance is denoted by *p ≤ 0.05, **p ≤ 0.01, ***p ≤ 0.001.

## 3 Results

### 3.1 Evaluation of Mesothelin expression on cervical cancer

**Figure 1:**
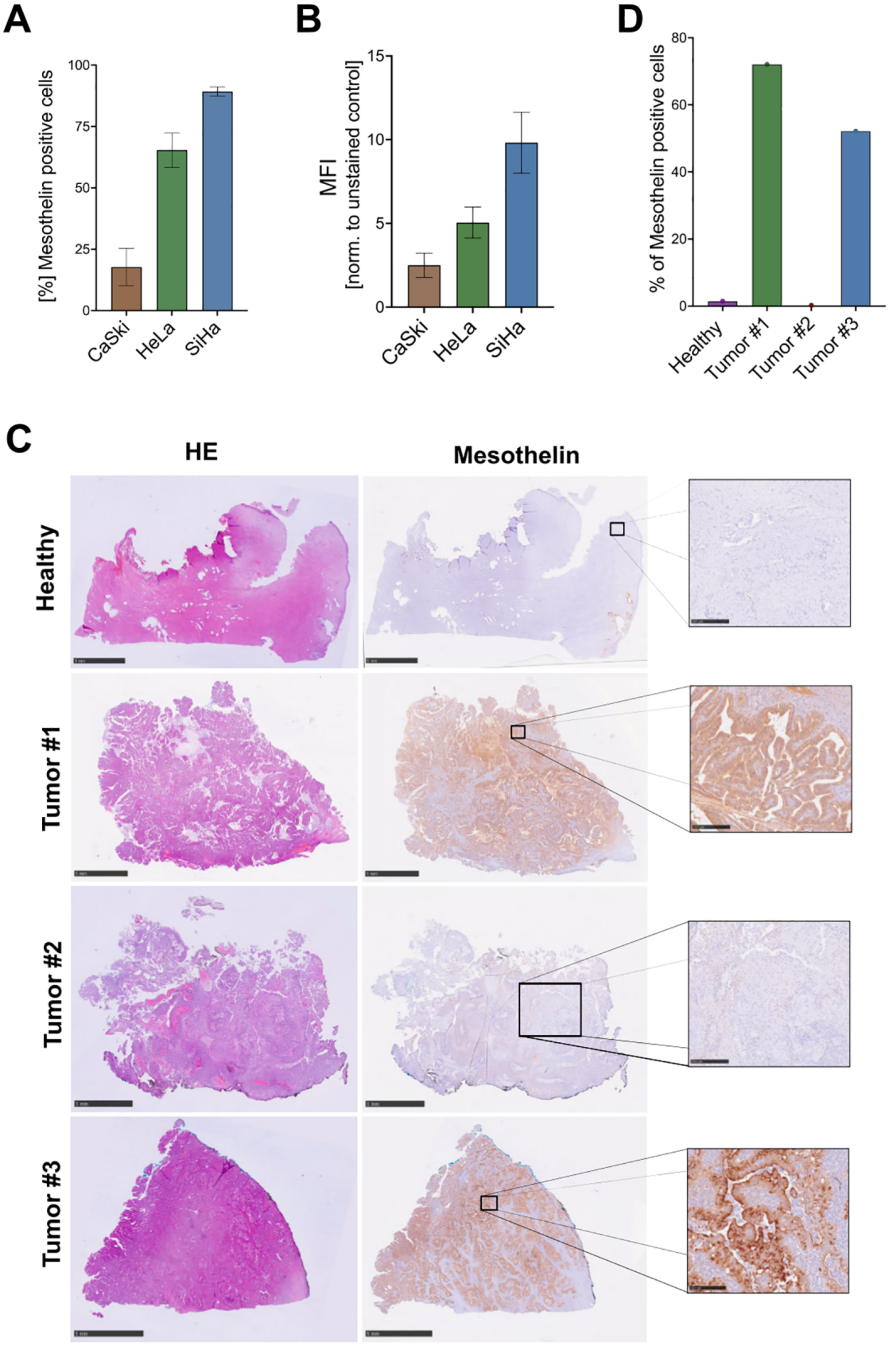
Detection of Mesothelin expression on cervical cancers. Cervical cancer cell lines were screened for cell surface expression of Mesothelin by flow cytometry to quantify the percentage of Mesothelin^+^ cells **(A)**, and the level of expression, here depicted by mean fluorescence intensity (MFI) **(B)**. Data are displayed as mean ± SD (n=3) **(A, B)**. Immunohistochemical staining was performed on paraffin sections of primary cervical cancer tissue samples derived from different cervical cancer patients and one healthy control. Hematoxylin and eosin (HE) stainings were performed alongside stainings for Mesothelin **(C)**. Quantification of the Mesothelin-stained paraffin sections shown in **(C, D)**. Size bars correspond to 5 mm for the healthy tissue, Tumor #1, and Tumor #3 samples. Size bars correspond to 1 mm for Tumor #2 sample. Scale bars of the zoomed cut-out areas that are designated with boxes correspond to 250 µm.

Flow cytometry was used to evaluate Mesothelin surface expression on several cervical cancer cell lines that represent different disease entities, including cervical squamous cell carcinoma (SiHa, CaSki) and adenocarcinoma (HeLa) (**Figures 1A, B**; **Supplementary Figure 1**). Mesothelin was detected on the surface of 89% of SiHa, 65% HeLa cells and only 17% of CaSki cells.

Additionally, we investigated the Mesothelin expression on sections derived from primary cervical cancer tissues and from a non-cancerous healthy sample. The primary cervical cancers were derived from both adeno- (Tumor #1 and Tumor #3) and squamous cell carcinomas (Tumor #2). IHC stainings show low or absent Mesothelin expression on the healthy cervical tissue. In contrast, tissue sections derived from two cervical cancer patients (Tumor #1 and Tumor #3) showed high expression, while one tissue section (Tumor #2) showed low expression of Mesothelin (**Figures 1C, D**). Moreover, we screened these primary samples for the expression of several immune cell markers, including CD3, CD11b, CD19, CD31, CD45, CD56. Healthy cervical tissue showed low abundance of CD45^+^cells. Tumor #1 shows low abundance of all screened immune cell markers, with slightly higher abundance of CD3^+^, CD11b^+^, CD31^+^, and CD45^+^ in Tumor #2. Tissue sections of Tumor #3 showed high abundance of CD3^+^, CD11b^+^, CD31^+^, and CD45^+^ cells. All tissue samples displayed low levels of CD19^+^ and CD56^+^ cells (**Supplementary Figures 2–6**).

### 3.2 Development of CAR-NK-92 cells to specifically target Mesothelin

**Figure 2:**
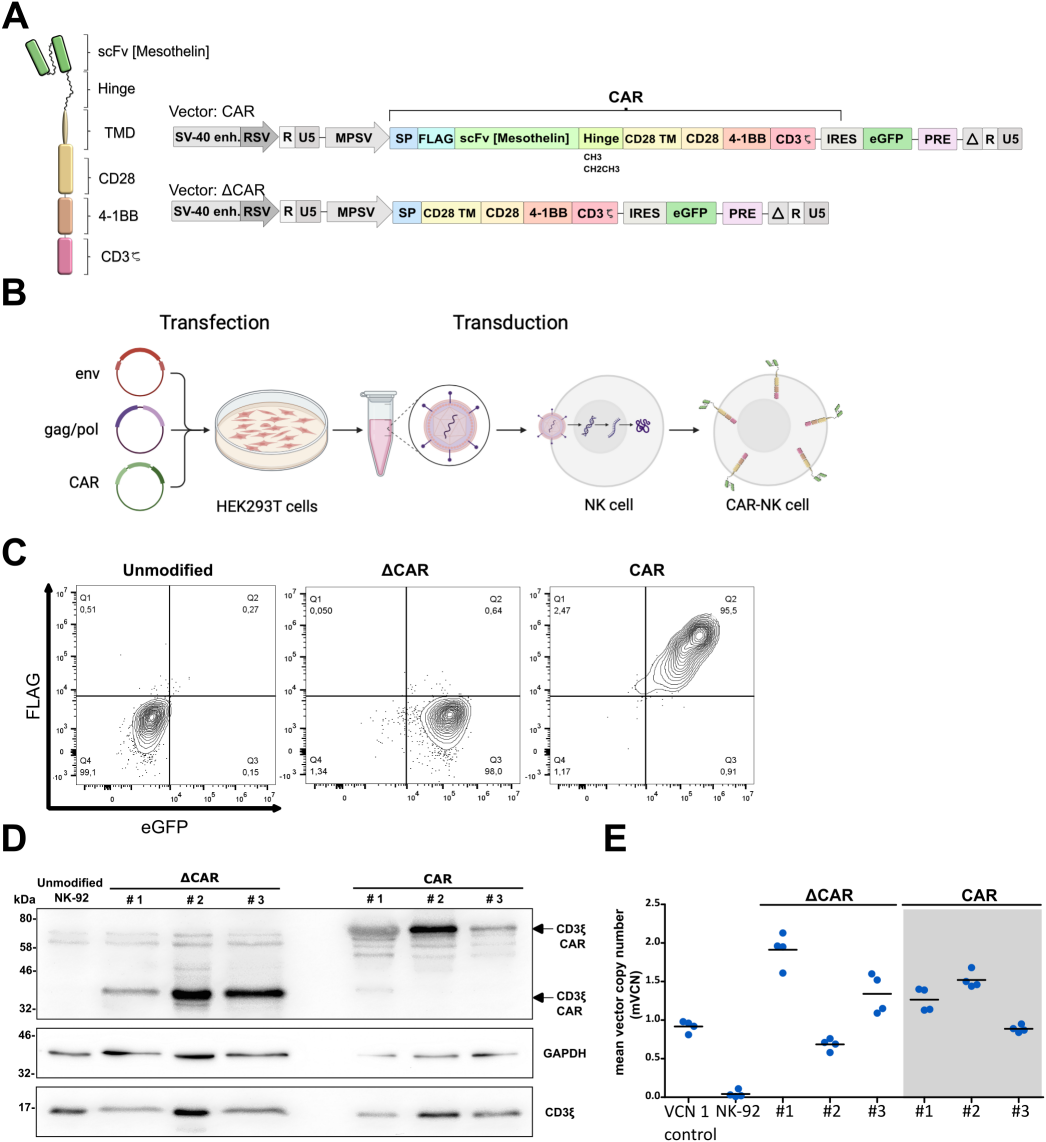
Generation and characterization of anti-Mesothelin CAR-NK-92 cells. The architecture of 3^rd^ generation CAR in self-inactivating (SIN) alpharetroviral vector plasmids for modification of NK-92 cells. Anti-Mesothelin single chain variable fragment (scFv) was combined with a long (CH2CH3) or midi (CH3) IgG1-derived hinge **(A)**. Key steps in production of modified CAR-NK-92 cells **(B)**. Analysis of CAR expression and vector integration via flow cytometry **(C)**, representative Western blot (n=2) **(D)** and mean vector copy number analysis in modified ΔCAR- and anti-Mesothelin CAR (midi hinge)-NK-92 cells (mean shown, n=4) **(E)**. CD3 midi-hinge CAR CD3ζ = 67 kDa, GAPDH = 38 kDa, endogenous CD3ζ = 17 kDa). The original images of the Western blots can be found in **Supplementary Figure 7**. Extracellular truncated CAR-NK-92 cells: ΔCAR. UTD: untransduced control NK-92 cells; VCN 1 control: induced pluripotent stem cell (iPSC) clone HD2 with a known VCN of 1 (25).

An alpharetroviral SIN vector was used to deliver an anti-Mesothelin-scFv (derived from the high-affinity HN1 antibody), an IgG1-based hinge [either CH3 midi hinge (m.h.) or CH2CH3 long hinge (l.h.)], a CD28-derived transmembrane domain, CD28 and 4-1BB co-stimulatory domains, and a CD3ζ signaling domain (**Figure 2A**). The main steps of CAR-NK-92 cell generation are shown in **Figure 2B**. The extracellular domain of the CAR was equipped with a FLAG-tag to ease CAR detection. In addition to unmodified NK cells, NK cells modified with an extracellular truncated CAR that lacks the scFv and hinge domains (=ΔCAR), served as a control CAR to investigate potential antigen-independent effects. After sorting, transduced NK-92 cells showed high eGFP (ΔCAR-NK-92 cells) and FLAG (Mesothelin CAR-NK-92 cells) levels, indicating a high purity of CAR-modified NK-92 cells (**Figure 2C**).

To exclude potential batch-effects, this procedure was repeated three times to create three independent batches of modified CAR-NK-92 cells. After sorting for FLAG^+^ eGFP^+^ modified anti-Mesothelin CAR-NK-92 cells and eGFP^+^ ΔCAR-NK-92 cells, the surface expression of CAR on the modified cells was routinely assessed via flow cytometry. The modified cells maintained their initial purity, with a positivity rate exceeding 90% for FLAG and eGFP, or eGFP exclusively in the case of truncated control (**Figure 2C**).

As another method to evaluate CAR expression in the three distinct batches of modified cells, whole cell lysates were analyzed by immunoblotting. CAR constructs were detected with anti-CD3ζ antibody, and signals at the anticipated molecular weight of 67 kDa for CARs containing the midi-hinge (CH3) were observed. Endogenous CD3ζ (17 kDa), typically expressed in NK-92 cells, and GAPDH (38 kDa) proteins served as internal loading controls (**Figure 2D**).

Considering potential risks associated with high integration events during cellular modification using retroviral vectors and consequent transformation events, the modified cells were further characterized by determining the vector copy number (VCN) as a quantification of the number of alpharetroviral vector insertions in the genome of the transduced cells. This determination involved assessing the ratio of the WPRE sequence, inherent to the alpharetroviral vector backbone, relative to PTBP2, a housekeeping control that is endogenously present in the genome. Notably, comparable levels of vector integration were observed across all CAR-NK-92 cells, with an average of 1 to 2 integrations per cell (**Figure 2E**).

### 3.3 Cytotoxic effect of engineered CAR-NK-cells on cervical cancer cells

**Figure 3:**
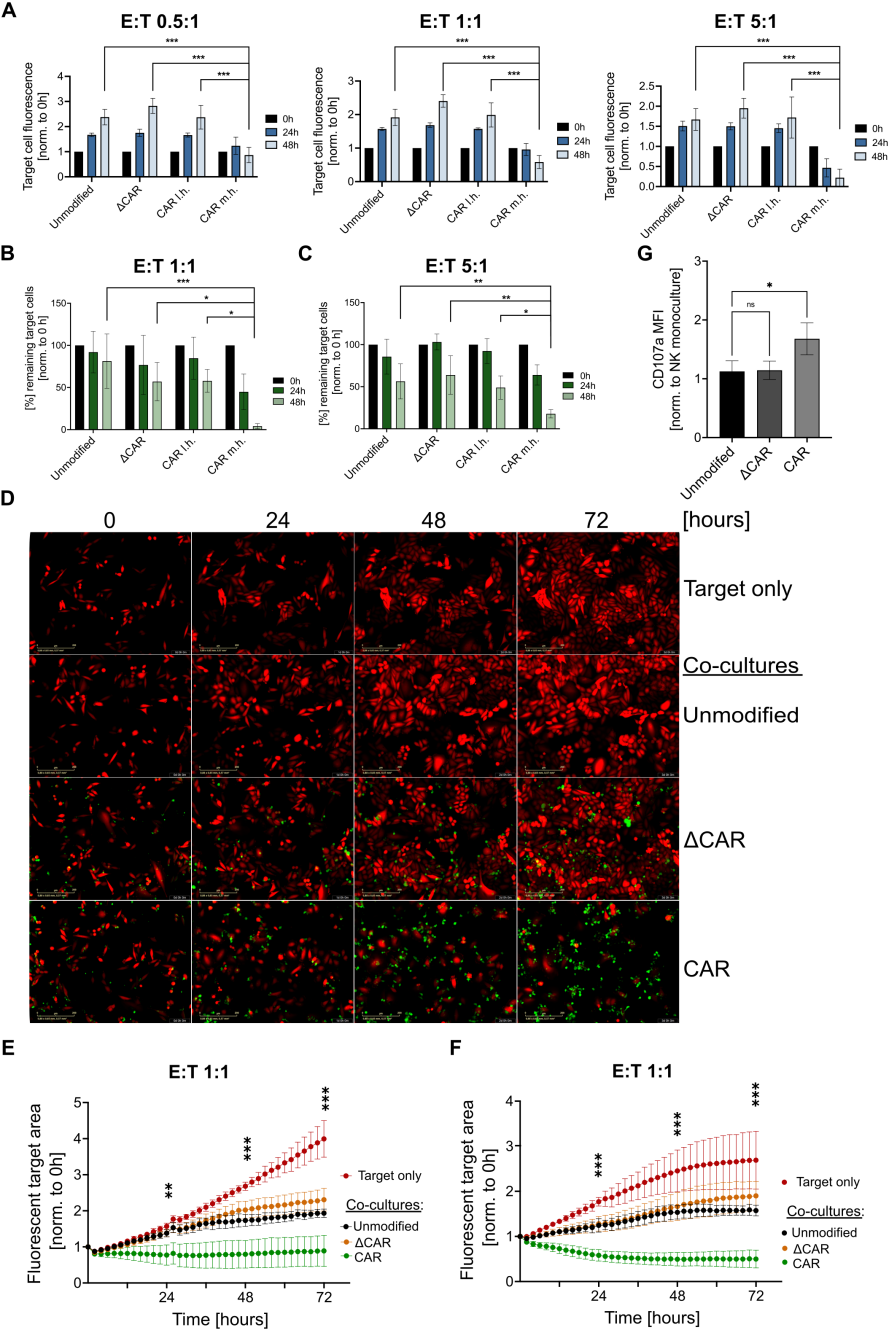
Anti-Mesothelin CAR-NK-92 cells efficiently eliminate cervical cancer cells. Analysis of CAR-NK-92 cell (effector) cytotoxicity against mCherry^+^ SiHa as quantified via Fluoroskan™ **(A)**. Flow cytometry-based cytotoxicity assays on mCherry^+^ SiHa **(B)** and mCherry^+^ HeLa **(C)** cells. Live-cell imaging of mCherry**^+^** SiHa cells (red) that were co-cultured with effector NK cells (green), are displayed at multiple timepoints (0h, 24h, 36h, 72h) after initial co-culture. Images were acquired using the Incucyte^®^ **(D)**. Quantification of the live-cell imaging cytotoxicity experiments is displayed for SiHa **(E)** and HeLa **(F)** using a 1:1 E:T ratio. Images showing the fluorescent target area were acquired every two hours for a total duration of 72 hours using the CELLCYTE X™. CD107a expression of NK-92 cells after 3 hours co-culture at 1:1 E:T ratio with mCherry^+^ SiHa target cells was assessed by flow cytometry **(G)**. All experiments were performed with three separate transduction batches of NK-92 cells. Extracellular truncated CAR-NK-92 cells: ΔCAR. m.h.: midi-hinge. l.h.: long-hinge. E:T = effector: target cell ratio. Statistical analysis was performed using one-way ANOVA **(G)** or two-way ANOVA **(A-C, E, F)** for comparison of anti-Mesothelin CAR-NK-92 cells (m.h.) to each group within the same time point **(A-C, G)** or **(E, F)** between anti-Mesothelin CAR-NK-92 cells (m.h.) and unmodified NK-92 cells at 24h, 48h and 72h (n=3). Live-cell imaging experiments comprised three independent experiments, with each batch assessed in technical triplicates. Statistical significance is denoted by *p ≤ 0.05, **p ≤ 0.01, ***p ≤ 0.001, lack of significance designated as ns. Data are displayed as mean ± SD, scale bar 400 µm.

Reporter cervical cancer cell lines (SiHa, HeLa) expressing the mCherry fluorescent protein were generated and used as target cells to investigate the cytotoxicity of the effector NK cells. Here, co-cultures were seeded in different effector-to-target (E:T) ratios (0.5:1, 1:1, 10:1) and the fluorescence of target cells was measured at multiple time points following the initial co-culture (0h, 24h, 48h) using the Fluoroskan™ reader (**Figure 3A**).

At all E:T ratios tested, anti-Mesothelin (m.h.) CAR-NK-92 cells significantly (p<0.001) outperformed the control NK-92 cells, leading to a clear reduction of mCherry^+^ SiHa cells. Noteworthy, very little differences were observed between the different batches of modified NK cells. To further validate these results, we assessed the cytotoxicity of the effector cells by flow cytometry. After 24 hours of co-culture, anti-Mesothelin m.h. CAR-NK-92 cells with a midi-hinge reduced the remaining target cells by over 50 percent (mean=44.9% remaining target cells). Moreover, anti-Mesothelin (m.h.) CAR-NK-92 cells nearly completely eradicated (mean=4.1% remaining target cells) the mCherry^+^ SiHa cells after 48 hours in a 1:1 E:T ratio. In contrast, control NK-92 cells led to only a slight decrease in the percentage of remaining target cells (**Figure 3B**). Similar results were also seen with HeLa cells that were co-cultured in a 5:1 E:T ratio with anti-Mesothelin m.h. CAR-NK-92 cells. A strong reduction of fluorescent HeLa target cells was most evident after 48 hours of co-culture (mean=17.8% remaining target cells). Interestingly, using this 5:1 E:T ratio, HeLa cells were reduced by nearly 50 percent after 48 hours of co-culture with unmodified NK cells (mean=56.3% remaining target cells) (**Figure 3C**).

To visualize the previously observed NK-92 cell cytotoxicity and to investigate the kinetics of the immune-cell killing, we performed additional live-cell imaging experiments in a 1:1 E:T ratio. Based on the Flouroskan™- and flow cytometry-based cytotoxicity assays, we focused all subsequent investigations on the m.h. CAR construct. Upon initial co-culture, a series of images were acquired via live-cell imaging every two hours for a total duration of 72 hours, with morphological changes upon co-culture shown for SiHa co-cultures in **Figure 3D**. Quantification of these live-cell imaging experiments showed continuous proliferation of target cells in the monoculture setting, which is less prominent upon co-culture with unmodified NK-92 and ΔCAR-NK-92 cells. In contrast, co-culture of anti-Mesothelin CAR-NK-92 cells led to a significant reduction of the mCherry^+^ SiHa cells (**Figure 3E**) and HeLa cells (**Figure 3F**) compared to unmodified NK-92 cells. These analyses showed that the cytotoxic activity of anti-Mesothelin CAR-NK-92 cells against the cervical cancer cells was initiated early after initiating co-culture (**Figures 3E, F**). Co-culture experiments showed functional cytotoxicity of anti-Mesothelin CAR-NK-92 cells by significantly increased CD107a cell surface levels as compared to non-transduced control NK-92 cells (p=0.03). In contrast, ΔCAR-NK-92 cells did not show significant changes of CD107a levels on the cell surface after target cell exposure (p=0.99) (**Figure 3G**).

As the configuration of the signaling domain can influence the activity of CAR-modified cells, we compared the activity of anti-Mesothelin CAR-NK-92 cells engineered with the 3^rd^-generation CAR construct with those modified with 2^nd^-generation CAR constructs. Quantitative analysis of live-cell imaging cytotoxicity assays demonstrated that 2^nd^- and 3^rd^-generation anti-Mesothelin CARs induced comparable levels of target cell lysis over time (**Supplementary Figure 8**).

### 3.4 Challenging Mesothelin antigen specificity of generated CAR-NK-92 cells

Antigen specificity of CAR-NK cells towards the target antigen is clearly a key aspect for successful implementation of this approach. To assess the antigen specificity of the anti-Mesothelin CAR-NK-92 cells, we performed a CRISPR-Cas9-mediated knockout of Mesothelin in SiHa cells using two different ATTO-labelled gRNAs that target distinct loci of the Mesothelin coding sequence. A high ATTO-fluorescence showed an efficient nucleofection of SiHa cells (**Supplementary Figure 9A**).

**Figure 4:**
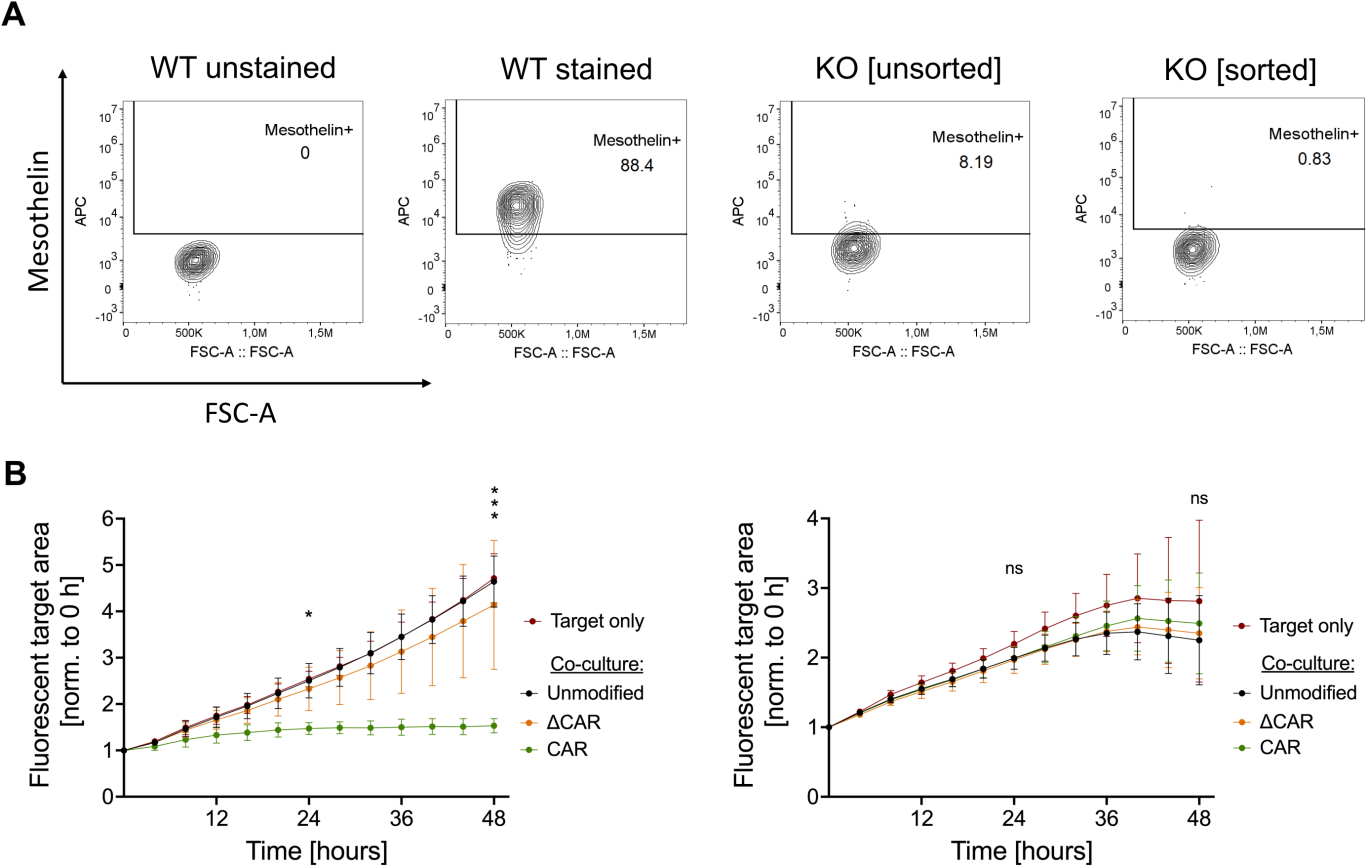
Investigation of anti-Mesothelin CAR-NK-92 cell specificity against Mesothelin^-^ cervical cancer cells. Mesothelin cell surface expression was analyzed by flow cytometry on wild-type (WT), non-sorted Mesothelin-knockout (KO), and sorted Mesothelin-KO SiHa cells **(A)**. Cytotoxicity of unmodified NK-92, ΔCAR-NK-92, and anti-Mesothelin CAR-NK-92 cells was evaluated by live-cell imaging (Sartorius Incucyte^®^) in a 1:1 E:T ratio against wild-type SiHa cells (left graph) and Mesothelin^-^ KO SiHa cells (right graph) **(B)**. Statistical analysis compares unmodified with anti-Mesothelin CAR-NK-92 cells. Significance is denoted by *p ≤ 0.05, ***p ≤ 0.001, lack of significance designated as ns. Data are displayed as mean ± SD, n=3. Extracellular truncated CAR-NK-92 cells: ΔCAR.

In line with previous experiments (**Figure 1A**), non-modified SiHa cells were highly positive for Mesothelin expression (88.4%), while the expression was strongly reduced to 8.2% in Mesothelin-knockout SiHa cells. The CRISPR-Cas9-modified SiHa cells were then sorted to obtain a population of Mesothelin^-^ cells (**Figure 4A**). The knockout was further analyzed on the DNA-level by targeted sequencing, showing effective disruption of the Mesothelin target sequence (**Supplementary Figures 9B-D**). Mesothelin^-^ target cells were stained with a red-fluorescent cell tracker prior to monitoring them during the co-culture experiments. While anti-Mesothelin CAR-NK-92 cells outperformed the unmodified and ΔCAR-NK cells against antigen-positive target cells, this effect was abolished upon co-culture of antigen negative cells, supporting specificity of the anti-Mesothelin CAR-NK-92 cells towards this target antigen (**Figure 4B**; **Supplementary Figure 10**).

### 3.5 Efficiency of anti-Mesothelin CAR-NK-92 cells against the cervical cancer spheroid model

**Figure 5:**
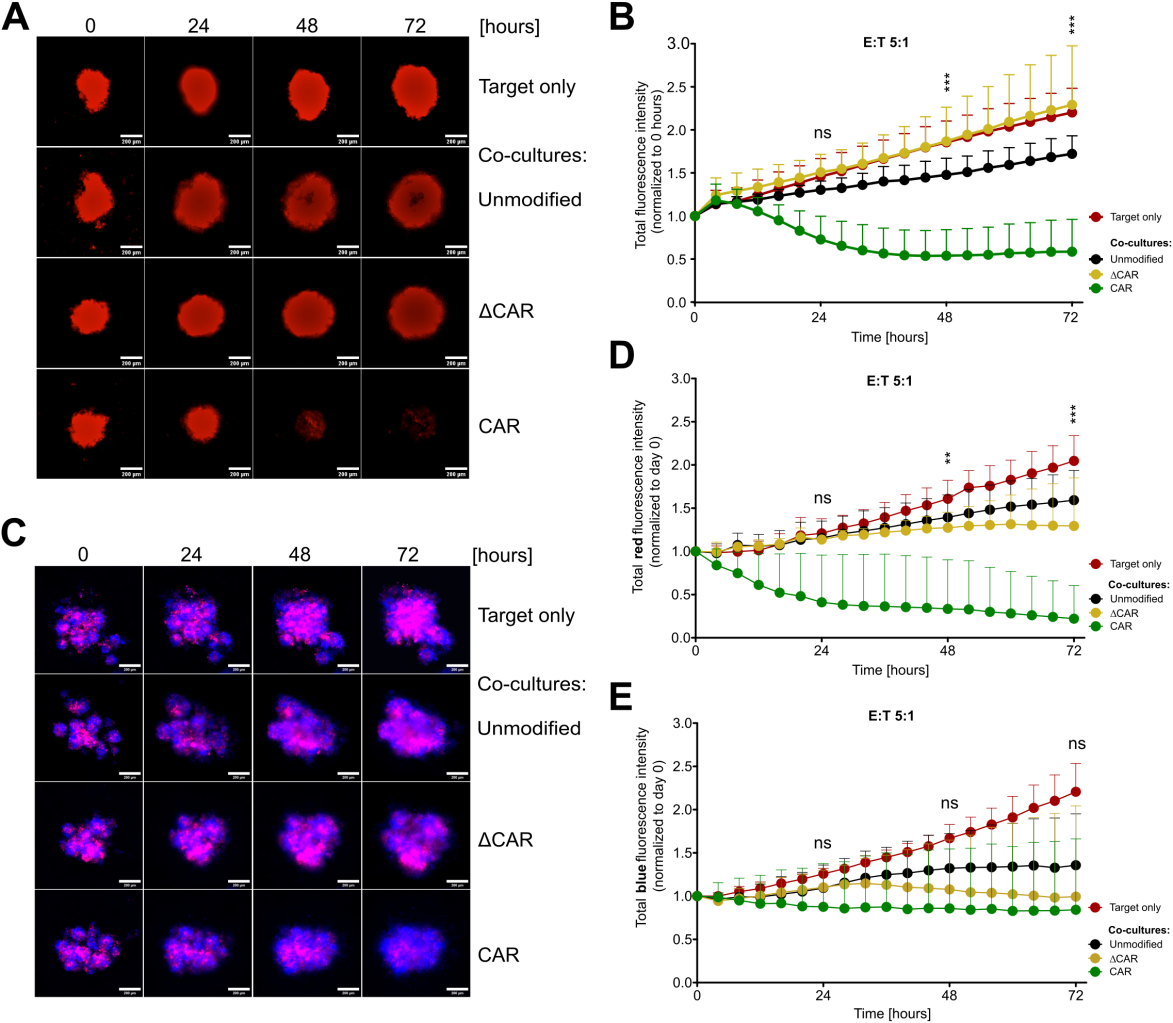
Efficient eradication of 3D tumor spheroids upon co-culture with anti-Mesothelin CAR-NK-92 cells. Fluorescent images captured during live-cell imaging-based cytotoxicity assays depict spheroids derived from mCherry^+^ Mesothelin^+^ SiHa cells **(A, B)** or from mixtures of mCherry^+^ Mesothelin^+^ and BFP^+^ Mesothelin^-^ SiHa cells **(C-E)** co-cultured with NK-92 cells at a 5:1 effector-to-target (E:T) ratio. Images were acquired every four hours over 72 hours. Total red fluorescent intensity (indicative of mCherry^+^ Mesothelin^+^ SiHa cells) **(B, D)** and total blue intensity (indicative of BFP^+^ Mesothelin^-^ SiHa cells) **(E)** were quantified and normalized to day 0 using CELLCYTE X™ software. Mean values with standard deviations are presented in the graphs. Statistical significance was determined using two-way ANOVA comparing anti-Mesothelin CAR-NK-92 (CAR) cells with unmodified NK-92 cells at 24 h, 48 h, and 72 h of co-culture. The study comprised three independent experiments, each including three separate NK-92 transduction batches, with each batch assessed in technical triplicate. Statistical significance is denoted by **p ≤ 0.01, ***p ≤ 0.001, lack of significance designated as ns. Data are displayed as mean ± SD (n=3). Scale bar 200 µm. Extracellular truncated CAR-NK-92 cells: ΔCAR.

Similar to conventional cytotoxicity assays conducted on 2D monolayers, we established cytotoxicity assays on mCherry-expressing SiHa spheroids in 96-well plates using an automated live imaging analyzer, enabling real-time monitoring of the kinetics of CAR-NK-92 cell activity against tumor spheroids. From 24 hours of co-culture onwards, noticeable morphological alterations in terms of size, shape, and compactness of the spheroids were observed in spheroids exposed to anti-Mesothelin CAR-NK-92 cells compared to untreated spheroids or those co-cultured with control cells. These differences became more pronounced at later time points. Starting from 48 hours, spheroids exposed to anti-Mesothelin CAR-NK-92 cells became smaller and lost their round shape and compactness, while spheroids treated with control cells exhibited only minor changes in compactness and tended to increase in size (**Figure 5A**). The total mCherry fluorescence intensity from the mCherry^+^ spheroids was considered as a parameter for describing spheroid density. The analyses revealed a continuous increase in total fluorescence intensity of untreated spheroids and those co-cultured with control effector cells. However, spheroids treated with anti-Mesothelin CAR-NK-92 cells exhibited a significant decrease (up to 46%) in total fluorescence intensity starting from 24 hours of co-culture, indicating a strong disruption of spheroid compactness due to the efficient elimination of mCherry^+^ cervical cancer cells (**Figure 5B**). These findings strongly support the enhanced killing capabilities of the anti-Mesothelin CAR-NK-92 cells, as previously observed in 2D *in vitro* assays (**Figure 3**). Furthermore, the obtained results underscore the efficacy of these cells even in 3D cell culture conditions.

To further assess the specificity of anti-Mesothelin CAR-NK-92 cells, we conducted experiments using mixed spheroid cultures. SiHa cells expressing mCherry and Mesothelin (Mesothelin^+^) were mixed with SiHa cells expressing blue fluorescent protein (BFP) and lacking Mesothelin (Mesothelin^-^) at a 1:1 ratio to form stable spheroids. These mixed spheroid cultures were then treated with anti-Mesothelin CAR-NK-92 cells, ΔCAR-NK-92 cells, or unmodified NK-92 cells for 72 hours. BFP and mCherry fluorescence signals were monitored and quantified using automated live-cell imaging. After just 24 hours of co-culture, a reduction in the total intensity of the mCherry fluorescence signal was observed (**Figures 5C, D**) and became more pronounced at later time points, supporting the results obtained from monoculture spheroids of mCherry^+^ cells (**Figures 5A, B**; **Supplementary Figure 11**).

In contrast, the total intensity of the BFP fluorescence signal, which originates from the Mesothelin^-^ SiHa cells, showed minimal decrease (95% of the initial value) over the course of the co-culture with anti-Mesothelin CAR-NK-92 cells (**Figure 5E**). The stability of BFP signal in Mesothelin^-^ cells further underscores the targeted action of anti-Mesothelin CAR-NK-92 cells, confirming their potential efficacy in selectively targeting Mesothelin-expressing tumor cells while sparing non-expressing cells.

### 3.6 Synergistic effect of CAR-NK-92 cells and cisplatin chemotherapy

**Figure 6:**
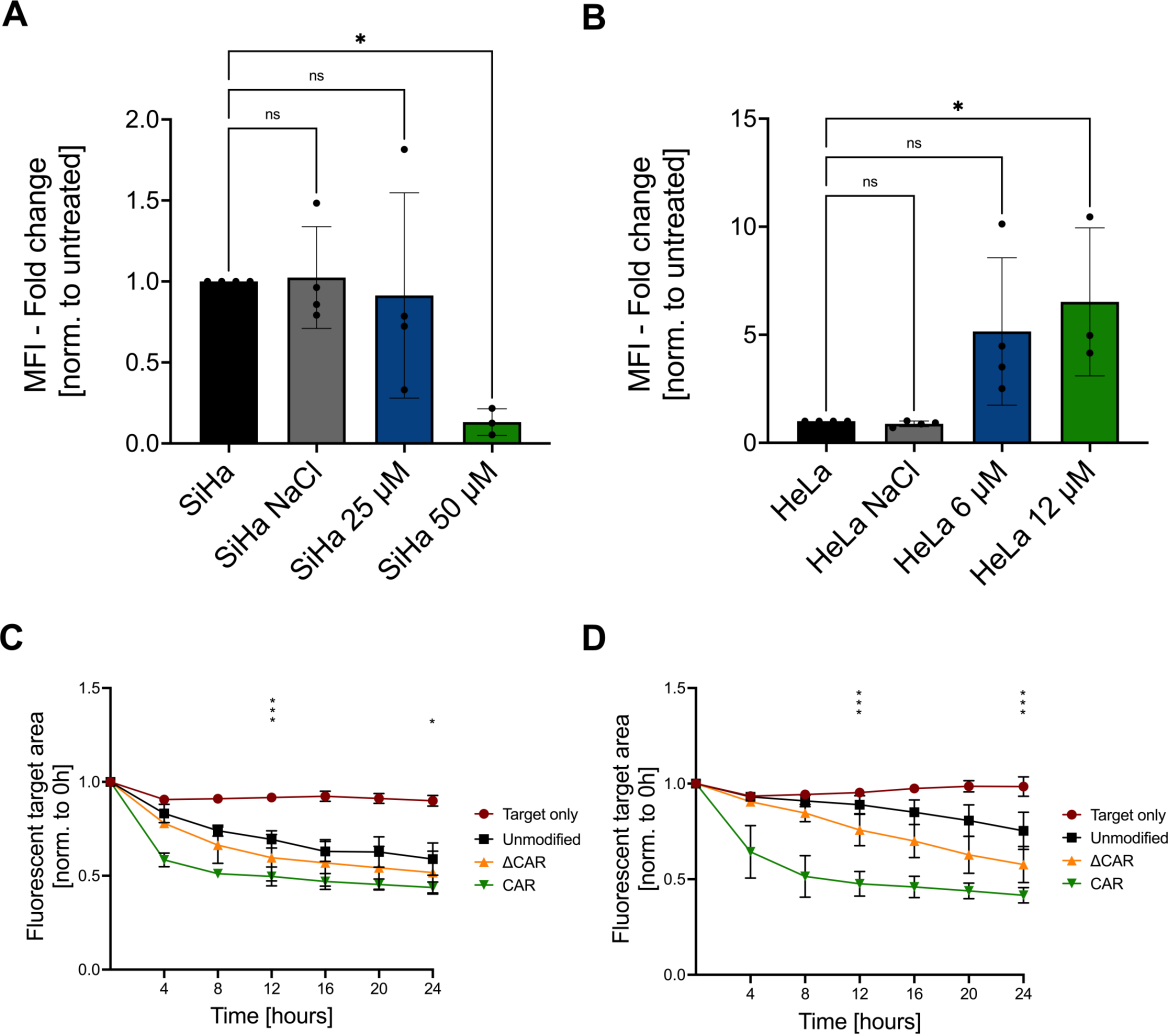
Combination of CAR-NK-92 cells with chemotherapy. Flow cytometry was used to analyze the mean fluorescence intensity (MFI) of a Mesothelin staining on SiHa cells **(A)** and HeLa cells **(B)** with or without pretreatment with cisplatin or solvent control (NaCl) (n=3). Live-cell imaging (CELLCYTE X™) was used to evaluate the cytotoxicity of unmodified NK-92, ΔCAR-NK-92, and anti-Mesothelin CAR-NK-92 (CAR) cells co-cultured in a 1:1 E:T ratio with SiHa **(C)** and HeLa **(D)** cells that were pre-treated with IC50 values of cisplatin for 72 hours (n=3). Statistical analysis compares unmodified with anti-Mesothelin CAR-NK-92 cells. Significance is denoted by *p ≤ 0.05, ***p ≤ 0.001, lack of significance designated as ns. Data are displayed as mean ± SD. Norm.: normalized. Extracellular truncated CAR-NK-92 cells: ΔCAR.

As cisplatin continues to be a main component of treatment regimens for cervical cancer, it is of interest to identify novel approaches, such as anti-Mesothelin CAR-NK-92 cells, that can be applied in combination with cisplatin with the aim to potentiate the elimination of cervical cancer cells. SiHa and HeLa cells were incubated for 72 hours with several cisplatin concentrations and the respective IC50 values were determined to be 25 µM (SiHa) and 6 µM (HeLa). Flow cytometry analyses showed that cisplatin treatment of SiHa cells significantly reduced the Mesothelin expression dose-dependently as shown by reduced MFI levels (**Figure 6A**). In contrast, treatment of HeLa cells with cisplatin led to up to a five-fold increase of the Mesothelin expression (**Figure 6B**). To investigate the cytotoxicity of anti-Mesothelin CAR-NK cells against cisplatin pre-treated cervical cancer cells, we performed co-cultures and monitored the remaining target cells by live-cell imaging. Compared to non-cisplatin-treated cervical cancer cells (**Figure 3**), we observed an increased cytotoxicity of unmodified NK-92 cells against cisplatin pre-treated target cells (**Figures 6C, D**). Moreover, we observed increased cytotoxicity of anti-Mesothelin CAR-NK-92 cells against cisplatin pre-treated SiHa cells, with significant differences compared to unmodified NK cells, especially during the early time points of the co-culture (**Figure 6C**). This effect was more prominent against cisplatin-pretreated HeLa cells. Compared to unmodified NK-92 cells, anti-Mesothelin CAR-NK-92 cells significantly reduced the fluorescent target area throughout the 72 hours of co-culture (**Figure 6D**).

### 3.7 Primary anti-Mesothelin CAR-NK cells are active against cervical cancer

**Figure 7:**
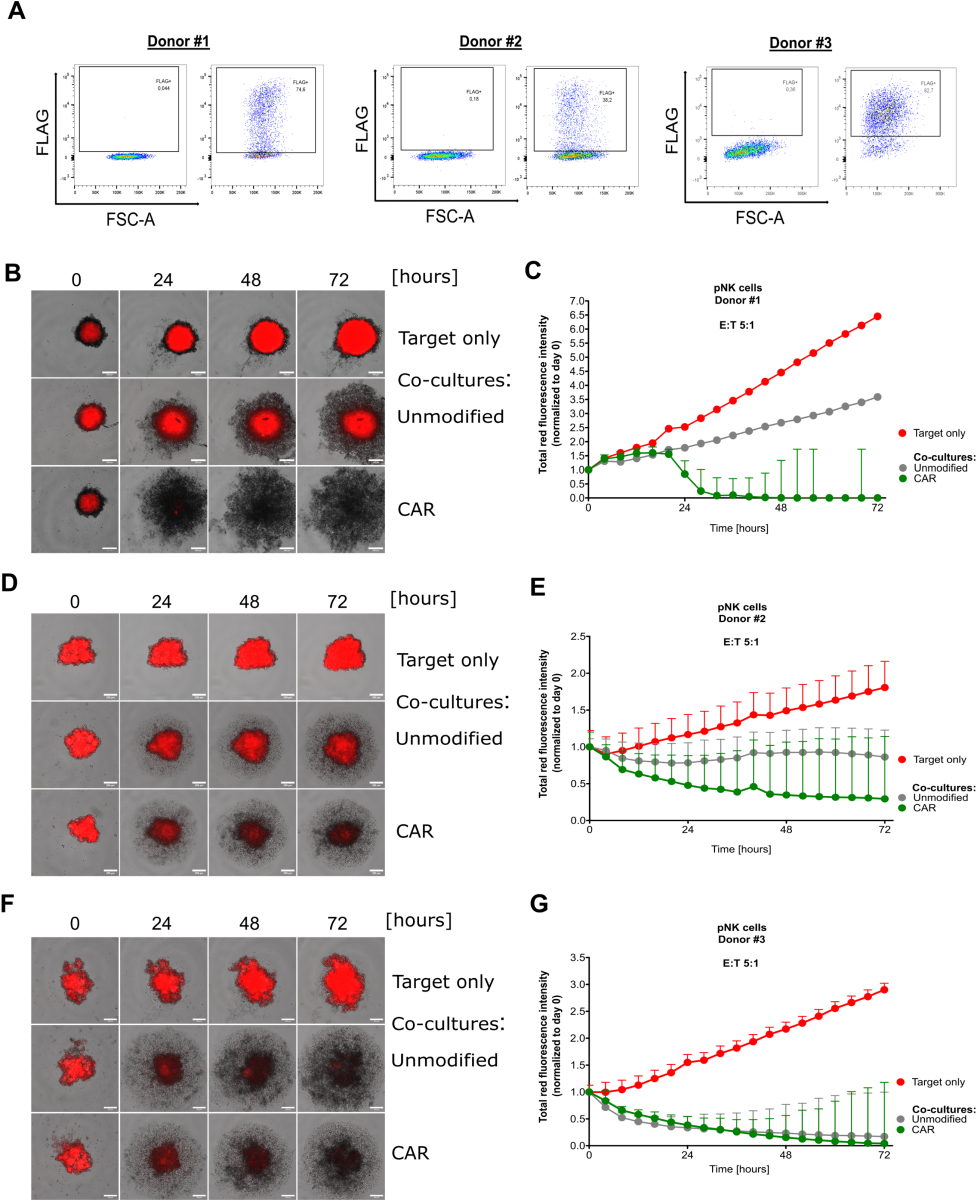
Primary NK cells efficiently eliminate cervical cancer cells in a 3D model. Investigation of cord blood-derived primary NK cells that have been double-transduced with viral supernatant encoding for anti-Mesothelin CARs. FLAG expression was assessed by flow cytometry for primary NK cells derived from three donors **(A)**. Fluorescent images captured during live-cell imaging-based cytotoxicity assays depict spheroids derived from mCherry^+^ SiHa cells co-cultured with primary NK cells at a 5:1 effector-to-target (E:T) ratio **(B, D, F)**. Total red fluorescent intensity (indicative of mCherry^+^ SiHa cells) quantified and normalized to day 0 using the CELLCYTE X™ software **(C, E, G)**. Data are displayed as mean ± SD. Scale bar 200 µm. Each graph depicts the results from an independent pNK donor accomplished in five technical replicates, n=1.

As a next step, we investigated whether our observations can be transferred into the use of primary cord blood-derived NK (pCB-NK) cells. Therefore, we isolated pCB-NK cells from cord blood-derived mononuclear cells using MACS and evaluated CD3 and CD56 expression on the isolated cells by flow cytometry. MACS-isolated cells showed nearly exclusively CD3^-^ and CD56^+^ cells, indicating a pure population of pCB-NK cells (**Supplementary Figures 12A, B**). To improve vector titers and pCB-NK cell transduction efficiency, we decreased the vector size by removing the IRES-eGFP expression cassette and transduced pCB-NK cells with the CAR-encoding alpharetroviral vectors (MOI = 30). Transduction efficiencies of pCB-NK cells varied among different donors, and were 74.6%, 38.2% and 82.7% for Donors #1, #2 and #3, respectively (**Figure 7A**). Anti-Mesothelin CAR-pCB-NK cells were directly used for cytotoxicity assays.

In the spheroid model cytotoxicity assay, unmodified control pCB-NK cells and anti-Mesothelin-CAR-pCB-NK cells were co-cultured with mCherry^+^ SiHa-derived spheroids for 72 hours. Automated live-cell imaging and quantification of the red fluorescent signal indicated that anti-Mesothelin CAR-pCB-NK cells from Donors #1 and #2 strongly inhibited the growth and stability of SiHa spheroids (**Figures 7B–E**). These findings are consistent with cytotoxicity results that we observed for the CAR-NK-92 cell line. However, as shown by the cytotoxicity data obtained with pCB-NK cells from Donor #3, some donor NK cells have inherently higher activities that are not enhanced by introduction of the anti-Mesothelin CAR (**Figures 7F, G**), indicating donor-specific differences.

To directly compare the effect of CAR-modified T- and NK cells against cervical cancer cells, we isolated primary NK (pPB-NK) and T (pPB-T) cells from PBMCs from the same donors and evaluated CD3 and CD56 expression profiles for NK cells and CD4 and CD8 expression for T cells (**Supplementary Figures 12B, C**). Subsequently, pPB-T and pPB-NK cells were transduced with alpharetroviral vectors encoding for the anti-Mesothelin CARs. Transduction efficiencies of pPB-NK cells were 49.5% (Donor #1) and 48.3% (Donor #2) (**Supplementary Figures 13A, B**). Compared to pPB-NK cells, pPB-T cells showed lower transduction efficiencies with 17.4% and 12.9% FLAG-positivity, with slightly higher transduction of CD4^+^ pPB-T cells compared to CD8^+^ pPB-T cells (**Supplementary Figures 13C, D**). To assess cytotoxicity, we co-cultured CAR-pPB-T and CAR-pPB-NK cells with cervical cancer spheroids in a live-cell imaging assay. Anti-Mesothelin CAR-pPB-T cells and CAR-pPB-NK cells both revealed strong cytotoxicity against Mesothelin^+^ SiHa cells, with anti-Mesothelin CAR-pPB-NK cells showing stronger target cell elimination and faster elimination kinetics. Moreover, unmodified pPB-NK cells revealed strong anti-cancer effects as compared to weaker activity of unmodified pPB-T cells (**Supplementary Figure 14A**). Additionally, we observed a strong decrease in the blue fluorescence signal of Mesothelin^-^ SiHa cells in co-cultures with unmodified and anti-Mesothelin CAR-pPB-NK cells. In contrast, unmodified pPB-T cells elicited limited cytotoxic activity against Mesothelin^-^ SiHa cells, and this cytotoxicity was enhanced for pPB-T cells that expressed the anti-Mesothelin CAR (**Supplementary Figure 14B**).

Overall, the same engineering approach can be successfully applied to NK cells derived from different sources further supporting broad applicability of our strategy to develop “off-the-shelf” NK cell-based immunotherapy.

## 4 Discussion

### 4.1 Need for immunotherapies

Conventional treatment options (surgery, radio-, chemotherapy) often fail in progressed cancer cases, with locally advanced cervical cancer reported to be recurrent of nearly one-third of patients (26). Furthermore, the 5-year survival rate for patients whose disease has spread to distant organs is less than 20 percent. Advancements in immunotherapies, including targeted antibodies that serve as immune check-point inhibitors (ICI) (*e.g.*, pembrolizumab) or that target the tumor vasculature (*e.g.*, bevacizumab), may eventually provide these patients with a better perspective. Treatment of cervical cancer with ICIs has shown promising clinical results if administered in combination with other therapy options (*e.g.*, chemotherapy, targeted drugs) (27, 28), as shown in the Keynote-826 study (NCT03635567). In addition to the success of ICIs, cellular immunotherapies (*e.g.*, CAR-based strategies) have the potential for long-term immunosurveillance and direct elimination of cancer cells (29). CAR-based strategies against cervical cancer have shown encouraging results in *in vitro* and *in vivo* studies (30–32), with many clinical trials being ongoing (5), NCT03356795, NCT04556669, NCT05518253, NCT05468190. Our data further indicate the suitability of CAR-based therapy approaches for solid cancers, as the anti-Mesothelin CAR-NK-92 cells showed strong anti-cervical cancer cytotoxicity in several 2D and 3D model systems.

### 4.2 Mesothelin as a promising target

The efficacy and safety of CAR-based immunotherapies are largely dependent on the selection of the target antigen, with tumor-specific antigens limiting the risk of on-target, off-tumor toxicities. Mesothelin is a promising target antigen as it is aberrantly expressed on cancer cells and has only limited expression in healthy adult tissues (33). Due to known difficulties to cultivate primary human cervical cancer cells, most studies rely on cell line models. Our data that showed high expression in HeLa cells and low expression in CaSki cells are in accordance with earlier reports (34–36), while studies investigating Mesothelin expression in SiHa cells are very limited. Interestingly, we observed low or no expression of Mesothelin in healthy cervical tissue sections, but high expression in most of the analyzed cervical cancer samples. A study that investigated 79 primary cervical cancer tissues identified high Mesothelin expression in the majority of cervical cancers, with higher expression levels being detected in adenocarcimonas (77%) than in squamous cell carcinomas (57%) (6). These results are in line with our results, which show higher intensities of Mesothelin staining on tissue samples that are derived from adenocarcinomas (Tumor #1 & Tumor #3). Taken together, the low expression profiles of Mesothelin in healthy adult tissue and high expression in most cervical cancers, make it a promising target antigen for immunotherapeutic approaches.

Apart from cervical cancer, Mesothelin has also been considered as a target for different malignancies. Cao et al. used lentiviral vectors to generate anti-Mesothelin CAR-NK cells for the treatment of gastric and ovarian cancer and identified the efficient elimination of target cells *in vitro* and *in vivo* (37, 38). A phase 1 clinical trial examined the treatment of cancer patients with recombinant anti-Mesothelin immunotoxin SS1P and found that 23 of 33 patients had a response or stable disease. Moreover, only limited side-effects were observed, with predominantly grade 1 and 2 (*e.g.*, hypoalbuminemia, fatigue), but no grade 4 toxicities (39). While over 30 clinical trials investigating Mesothelin CAR-T cells are registered on www.clinicaltrials.gov, only two investigate CAR-NK cells, and, thus far, none of these trials evaluate cervical cancer. Among the CAR-T trials, multiple studies investigate the use of anti-Mesothelin CAR-T cells against gynecological cancers, focusing mainly on ovarian cancer. Here, one study reported 2/3 patients with chemotherapy-refractory metastatic ovarian cancer treated with autologous anti-Mesothelin CAR-T cells achieved stable disease for 4.6 to 5.8 months, but no grade 3 or 4 adverse events (14). One phase 1 clinical trial evaluated the use of anti-Mesothelin CAR-T cells to treat patients with malignant pleural disease in combination with pembrolizumab. Over 68% of the patients had a response or stable disease, and the treatment was well-tolerated, with no on-target off-tumor toxicity observed (13).

### 4.3 NK vs T cell anti-cancer immunotherapy

However, despite these promising advances, some challenges directly associated with CAR-T cell therapies include graft-versus-host disease (GvHD), neurotoxicity, and cytokine release syndrome (CRS), which is caused by excessive release of inflammatory cytokines (1, 40–42). In contrast to CAR-T cells, application of CAR-NK cells carries a much lower risk for development of GvHD or CRS, with the latter likely due to a different profile of released cytokines (43–45). Application of cord blood-derived anti-CD19 CAR-NK cells resulted in overall response rates (ORR) of 73% in patients with CD19-positive lymphoid tumors, with most patients in complete remission in a Phase 1/2 clinical trial (44). Importantly, no neurotoxicity or CRS was detected. These data are in line with a recently published follow-up, which analyzed 37 patients with CD19^+^ B cell malignancies who were treated with anti-CD19 CAR-NK cells (46). Additionally, the natural capacity of NK cells to detect and eliminate cancer cells may help generate a greater elimination of tumor cells, including those that do not express the antigen targeted by the CAR, and thus better overcome the challenge of heterogeneity within solid tumors (47, 48). These CAR-independent, inherent anti-cancer effects of natural killer cells can be detected in our cytotoxicity assays using NK-92 and primary NK cells. Here, co-cultures of target cervical cancer spheroids with unmodified NK cells led to stronger elimination of target cells, as compared to T cells from the same donors in parallel assays. Consistently, anti-Mesothelin CAR primary NK cells also exhibited greater cytotoxic effects against Mesothelin^-^ and Mesothelin^+^ SiHa cells than CAR-T cells in the same experimental settings, indicating the dual nature of NK cells in the elimination of cancer cells (**Figures 3**–**7**; **Supplementary Figures 14A, B**). The observed enhanced cytotoxicity of anti-Mesothelin CAR-pPB-T cells over unmodified pPB-T cells against the Mesothelin^-^ cells might be explained by their proximity to Mesothelin^+^ target cells that caused hyperactivation of these immune cells, potentially leading to a bystander effect. In contrast, this effect was not evident in the NK cell experiments, likely due to the inherently high baseline cytotoxicity exhibited by unmodified NK cells (**Supplementary Figure 14B**). Our observations may reflect higher transduction efficiencies in CAR-NK cells that led to more robust CAR expression as well as differences in NK and T cell biology. These data are in line with previous studies, which demonstrated intrinsic cytotoxicity of NK cells against multiple malignancies (38, 49–52). However, some tumors might evade the intrinsic anti-cancer effects of NK cells *e.g.*, by upregulation of inhibitory molecules, or immunosuppressive tumor microenvironments (53). Thus, engineering NK cells with CARs is one strategy to ameliorate this clinical challenge.

### 4.4 CAR specificity in advanced tumor models

Although chimeric antigen receptor (CAR) cell therapy has demonstrated notable efficacy in treatment of hematologic malignancies [reviewed in (41)], extension of this approach to treat solid cancers revealed significant challenges, including the difficulty in identifying optimal target surface antigens. The lack of truly tumor-specific antigens contributes to on-target/off-tumor activity accompanied by potentially severe toxicities as engineered immune cells interact with normal healthy tissues (54, 55). Even when antigens with high tumor specificity are identified, their heterogeneous expression within tumors may allow antigen-negative tumor cells to evade selective CAR targeting (56). Consequently, there is a general need to identify novel tumor antigens that can be targeted to enhance the therapeutic potential of CAR cells against solid cancers by addressing concurrent challenges of specificity and heterogeneity.

Our study shows that CAR-NK-92 cells directed against Mesothelin are effective against cervical cancer cell models in *in vitro* assays (**Figures 3**, **5**, **6**). Moreover, knock-out of the target antigen Mesothelin on the same cancer cell lines was sufficient to escape CAR-mediated recognition and killing. While these results demonstrated strong specificity of the CAR-NK-92 cells to target Mesothelin-expressing cervical cancer cells, this data also suggest the presence of immune evasion mechanisms that lead to resistance of these tumor models to NK-92 and CAR-NK-92 cell therapy. Although this study demonstrated similar anti-tumor effects of 2^nd^ and 3^rd^ generation CARs in an *in vitro* setting with a limited co-culture duration (**Supplementary Figure 8**), this might be different in patients who are infused with the CAR-NK cells. Here, the additional co-stimulatory domains might benefit the activation and persistence of the CAR-NK cells and might promote interactions with other immune cells to orchestrate a combined immune response and hence might decrease the resistance of solid tumors to this therapeutic approach (57–60).

The structural organization, heterogeneous antigen expression and influence of the microenvironment in complex solid tumor tissues all represent critical challenges to successful treatment of solid tumors. One approach to try to model these important aspects of tumor biology is the generation of 3D tumor models, which are becoming more commonly used in preclinical studies of modern oncology (61, 62). In line with earlier reports (63), we successfully established spheroid models with cervical cancer cell lines. Notably, the anti-Mesothelin CAR-NK-92 cells generated in our study decreased spheroid integrity and eliminated cervical cancer cells even in this 3D model (**Figure 5**). These findings support the promise of our CAR-NK approach to overcome the complexities of solid tumor organization and the heterogeneous target antigen expression.

While cell line- and patient-derived spheroids may capture several aspects of tumor biology, precise pre-clinical (personalized) anti-cancer therapies demand consideration of the broader context of the tumor microenvironment (TME), including cellular heterogeneity, cell–cell interactions, extracellular matrix dynamics, and the presence of cancer stem cells. One commonly used approach is to use patient-derived xenograft (PDX) animal models. However, these models also have drawbacks, including lack of predictability for clinical trial outcomes, limitations in mimicking the complex process of human carcinogenesis, and increasing ethical concerns about the use of animal models (64). Nonetheless, important parameters such as CAR-NK cell trafficking to tumor sites, tumor infiltration, persistence, and off-target toxicities may be better evaluated with *in vivo* tumor models.

Cervical cancer organoids might offer a more personalized alternative as preclinical model systems for development of new immunotherapies since they possess a high potential to recapitulate key aspects of tumor biology while addressing some of the limitations associated with traditional models. The widespread use of cervical cancer organoids in preclinical immunotherapeutic studies is hampered by the challenges to successfully establish these organoid cultures (65–69).

### 4.5 Safety profile of our approach: vector choice and integration sites

The FDA recently raised additional safety concerns regarding CAR-T cell anti-cancer therapies due to the occurrence of malignancies after treatment of some patients (70, 71). While the overall number of these severe adverse events remains low, it is still important to address the potential for such side effects and to understand the underlying pathological mechanisms of these events with the aim to further lower the risk of therapy-related complications. Use of retroviral vector systems to integrate gene cargos such as CARs into the genome of the desired immune cell type (*e.g.*, T cells, NK cells, dendritic cells, macrophages) to allow long-term expression carries the inherent risk of insertional mutagenesis, which may inactivate tumor suppressor genes or activate oncogenes. One way to decrease the likelihood of these potential dangers is to limit the number of insertional events. A widely accepted practice for clinically used cells that were modified with retroviral vector technologies is to adhere to the upper limit of a mean of five vector insertions per therapeutic cell. Additionally, it is possible to screen CAR-NK-92 cell line cultures for a clone in which the CAR has integrated into a genomic safe harbor. Another main factor that influences the safety of retroviral vectors is the integration profile of the vector. Many completed and ongoing clinical trials employing CAR-T and -NK cells use gammaretroviral or lentiviral vectors to deliver and express the CARs in the respective cells. Gammaretroviral vectors have a preference for integration into transcription start sites and lentiviral vectors favor integration into transcriptional units. In contrast, we used self-inactivating (SIN) alpharetroviral vectors (19) to transduce NK-92 cells. Alpharetroviral vectors are known to have a more neutral integration pattern (72, 73), distinguishing them from the gammaretroviral and lentiviral vectors commonly used in clinical settings, and making alpharetroviral vectors potentially safer tools for human gene and cell therapy approaches.

### 4.6 Combinatorial trials as perspective - cisplatin

To clinically implement such novel CAR-based strategies, it might be beneficial to combine them with already available conventional treatment options. Such combinatorial treatments might be especially promising in the context of CAR-NK cells. Our results show an increased cytotoxicity of NK cells against cervical cancer cells that have been pre-treated with cisplatin (**Figure 6**). This indicates a sensitization of cervical cancer cells to NK-mediated killing upon cisplatin treatment, likely due to a potentiation of the natural anti-cancer effects of the NK-92 cells. Here, chemotherapeutic agents have been described to cause a stress-induced upregulation of NK cell-activation ligands, highlighting the potential for combining immunotherapeutic strategies that include NK cells with chemotherapy (74–77). To combine chemotherapy with CAR-based approaches, it is crucial to investigate the influence of the respective chemotherapeutic on the target antigen expression. We observed a differential regulation of the Mesothelin expression on SiHa and HeLa cells after cisplatin treatment, with reduced levels in SiHa cells and elevated levels in HeLa cells (**Figure 6**). Consequently, cisplatin-treated HeLa cells were more susceptible to anti-Mesothelin CAR-NK cell activity. Interestingly, Mesothelin was described to be correlated with chemo-resistance in ovarian cancer, and increased Mesothelin expression was detected in chemoresistant patients (78, 79). For such cases, pretreatment of cancers with cisplatin might sensitize them for a subsequent therapy using CAR-modified immune cells. As a result, the therapeutic sequelae, as well as the choice of the combination, might need to be tailored to distinct disease subtypes. Further studies should explore such correlations, with the aim to develop more effective therapeutic approaches to treat these patients.

## Data Availability

The raw data supporting the conclusions of this article will be made available by the authors, without undue reservation.
