# Corrigendum: Anti-Mesothelin CAR-NK cells as a novel targeted therapy against cervical cancer

**DOI:** 10.3389/fimmu.2025.1571555

**Published:** 2025-02-21

**Authors:** Ivana Kutle, Robert Polten, Jan Lennart Stalp, Jens Hachenberg, Felix Todzey, Ralf Hass, Katharina Zimmermann, Juliane von der Ohe, Constantin von Kaisenberg, Lavinia Neubert, Jan C. Kamp, Dirk Schaudien, Ann-Kathrin Seyda, Peter Hillemanns, Rüdiger Klapdor, Michael Alexander Morgan, Axel Schambach

**Affiliations:** ^1^ Institute of Experimental Hematology, Hannover Medical School, Hannover, Germany; ^2^ Department of Gynecology and Obstetrics, Hannover Medical School, Hannover, Germany; ^3^ Institute of Pathology, Hannover Medical School, Hannover, Germany; ^4^ Biomedical Research in Endstage and Obstructive Lung Disease Hannover (BREATH), German Center for Lung Research (DZL), Hannover, Germany; ^5^ Department of Respiratory Medicine and Infectious Diseases, Hannover Medical School, Hannover, Germany; ^6^ Fraunhofer Institute for Toxicology and Experimental Medicine, ITEM, Hannover, Germany; ^7^ Department of Gynecology and Obstetrics, Albertinen Hospital Hamburg, Hamburg, Germany; ^8^ Division of Hematology/Oncology, Boston Children’s Hospital, Harvard Medical School, Boston, MA, United States

**Keywords:** cervical cancer, immunotherapy, chimeric antigen receptor (CAR), CAR-NK cells, Mesothelin, chemotherapy, CAR-T cells

In the published article, there was an error in **Supplementary Figure 8**. The graphical data shown in **Supplementary Figure 8** was inadvertently duplicated, so that the same data is displayed twice in the figure. This error was present only in the submitted PDF version of the **Supplementary Material**, while the individually submitted figure files were correct. It appears that an intended edit was not properly incorporated into the final compiled PDF that was uploaded during submission, leading to this unintended duplication.

The correct **Supplementary Figure 8** can be found in the supplementary material link given in the publication. The legend to the correct **Supplementary Figure 8** appears below:

“**Supplementary Figure 8** Comparison of 2^nd^ and 3^rd^ generation CARs. Live cell imaging-based cytotoxicity assays of anti-Mesothelin CAR-NK-92 cells against mCherry^+^ SiHa cells. CAR-NK-92 cells were co-cultured with mCherry^+^ SiHa cells for 72 hours in a 96-well plate at 1:1 **(A)** and 1:16 **(B)** effector to target (E:T) ratios. The anti-tumor activity of the different generations of anti-Mesothelin CAR-NK-92 cells was determined by monitoring the changes in the red-surface area over 72 hours. Data are displayed as mean ± SD, n=3-4, conducted in technical triplicates. Statistical analysis was performed using two-way ANOVA for comparison of 28- BBζ: 3^rd^ generation anti-Mesothelin CAR-NK-92 cells (CD28-41BB-CD3ζ) to BBζ: 2^nd^ generation (41BB-CD3ζ) and 28ζ: 2^nd^ generation (CD28-CD3ζ) anti-Mesothelin CAR-NK-92 cells at 24, 48 and 72 hours. Non-significant differences are indicated as ns.”

The authors apologize for this error and state that this does not change the scientific conclusions of the article in any way. The supplementary material has been updated within the original article.

