# Supplementary material for: Anti-Mesothelin CAR-NK cells as a novel targeted therapy against cervical cancer
Source: Front Immunol. 2024 Dec 16;15:1485461. doi: 10.3389/fimmu.2024.1485461 (PMC11707549; doi:10.3389/fimmu.2024.1485461)
Supplement: Supplementary file 7 [file DataSheet1.pdf]

Supplementary Material

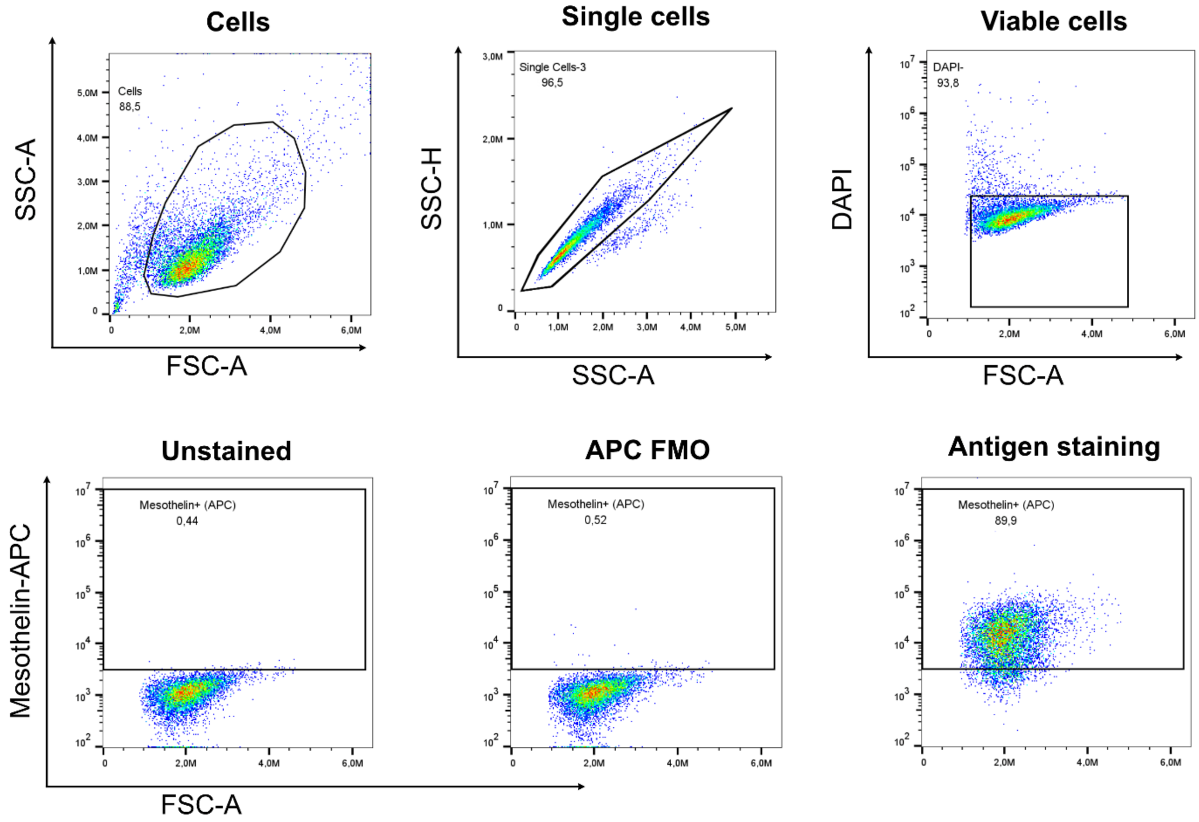

Supplementary Figure 1. Gating strategy to analyze Mesothelin surface expression. Cells were separated from cellular debris by FSC-A and SSC-A scatter plots. Single cell populations were determined by SSC-A and SSC-H and DAPI-negative cells were considered as viable. To assess the cell surface Mesothelin expression, gates were set based on the fluorescence minus one (FMO) sample that contained all stainings except for Mesothelin-APC.

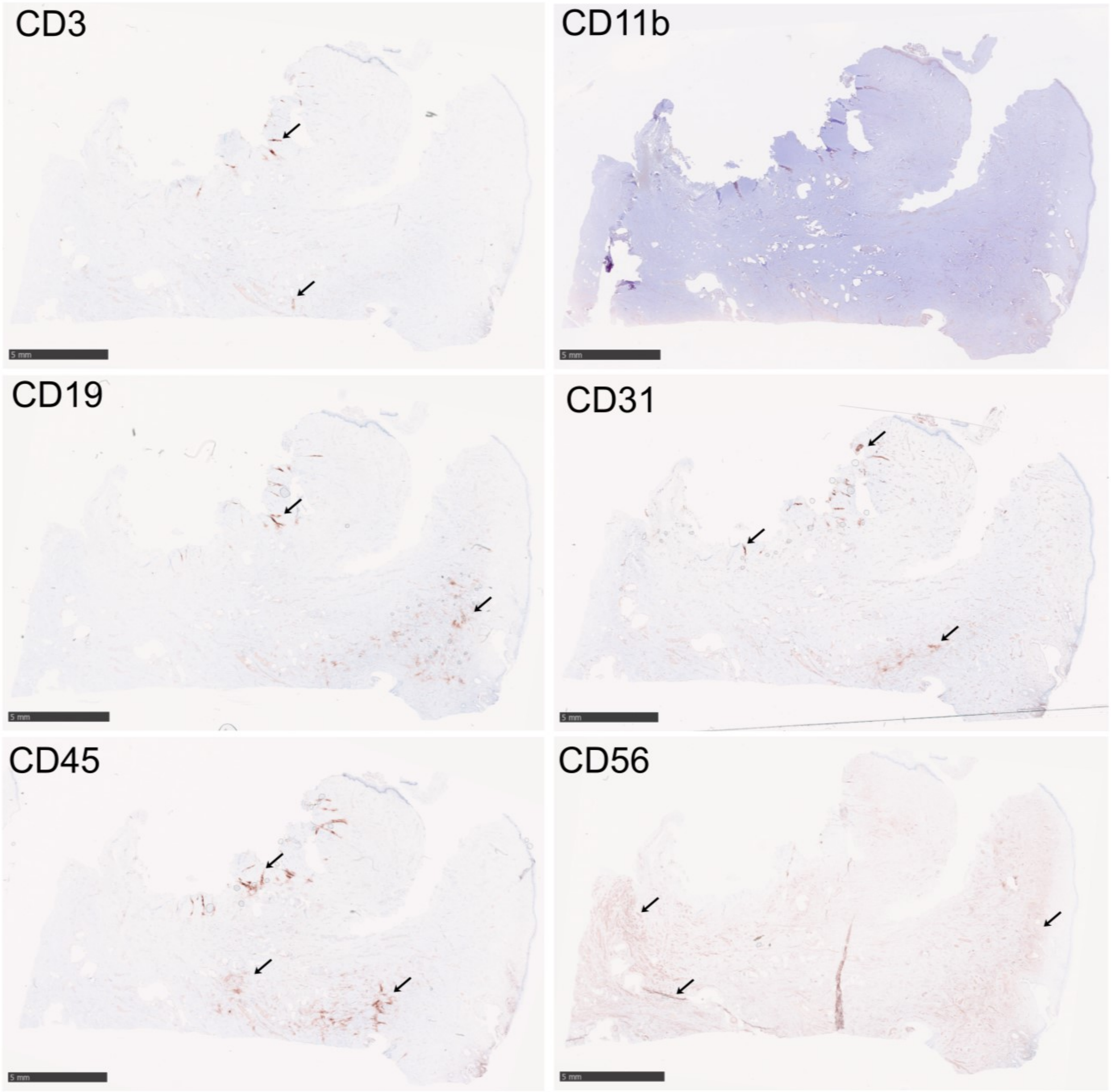

Supplementary Figure 2. Immunohistochemistry of healthy cervix tissue. Immunohistochemistry staining (brown signal) on non-cancerous tissue section show the expression of multiple markers to identify the presence of various cell types, including CD3 (*e.g.,* T cells), CD11b (*e.g.,* macrophages), CD19 (*e.g.,* B cells), CD31 (*e.g.*, endothelial cells), CD45 (*e.g.,* hematopoietic cells), CD56 (*e.g.,* natural killer cells). Scale bars correspond to 5 mm. Arrows indicate antigen-positive regions.

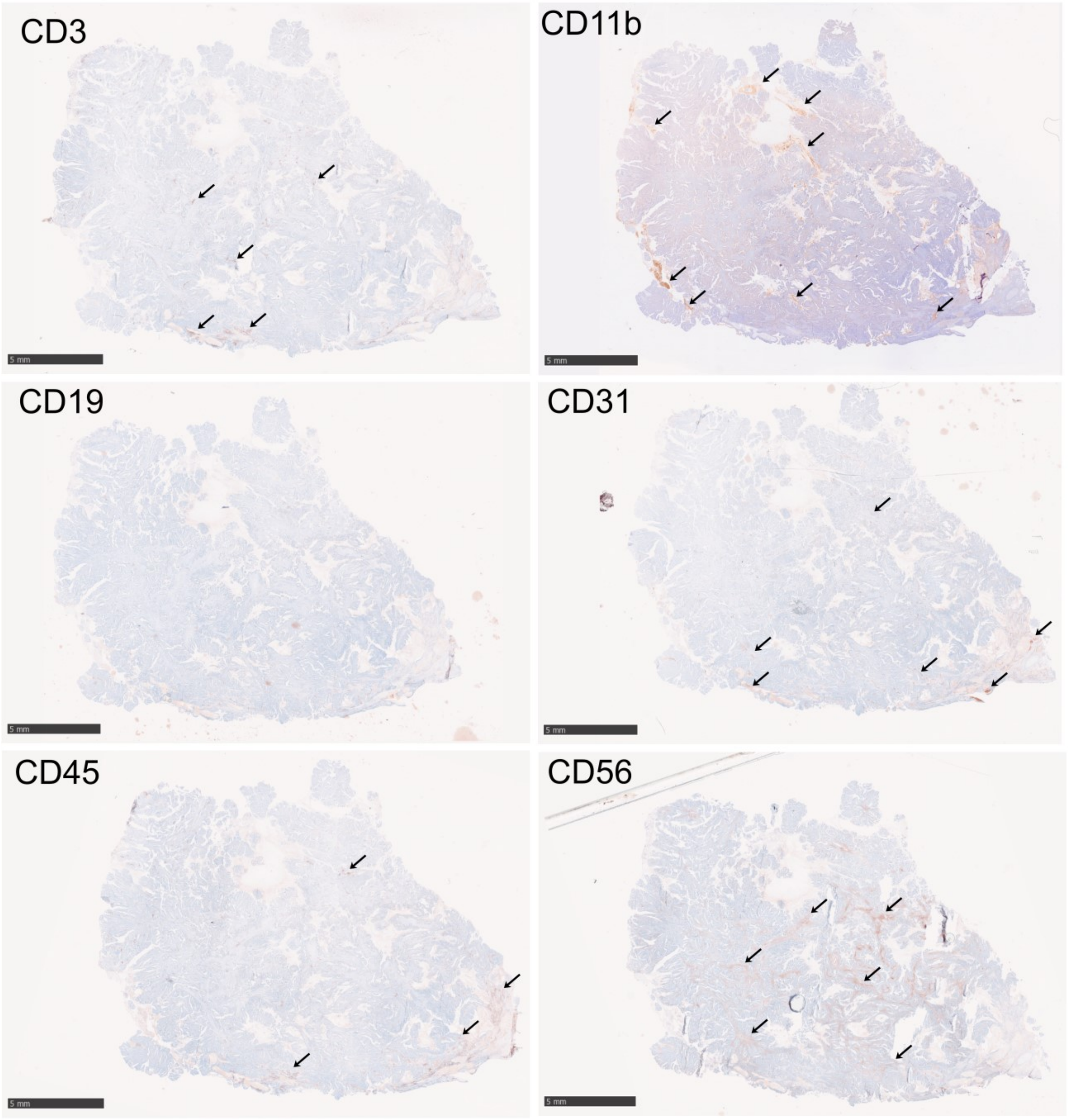

Supplementary Figure 3. Immunohistochemistry of Tumor #1. Immunohistochemistry staining (brown signal) on cervical cancer tissue sections show the expression of multiple markers to identify the presence of various cell types, including CD3 (*e.g.,* T cells), CD11b (*e.g.,* macrophages), CD19 (*e.g.,* B cells), CD31 (*e.g.*, endothelial cells), CD45 (*e.g.,* hematopoietic cells), CD56 (*e.g.,* natural killer cells). Scale bars correspond to 5 mm. Arrows indicate antigen-positive regions.

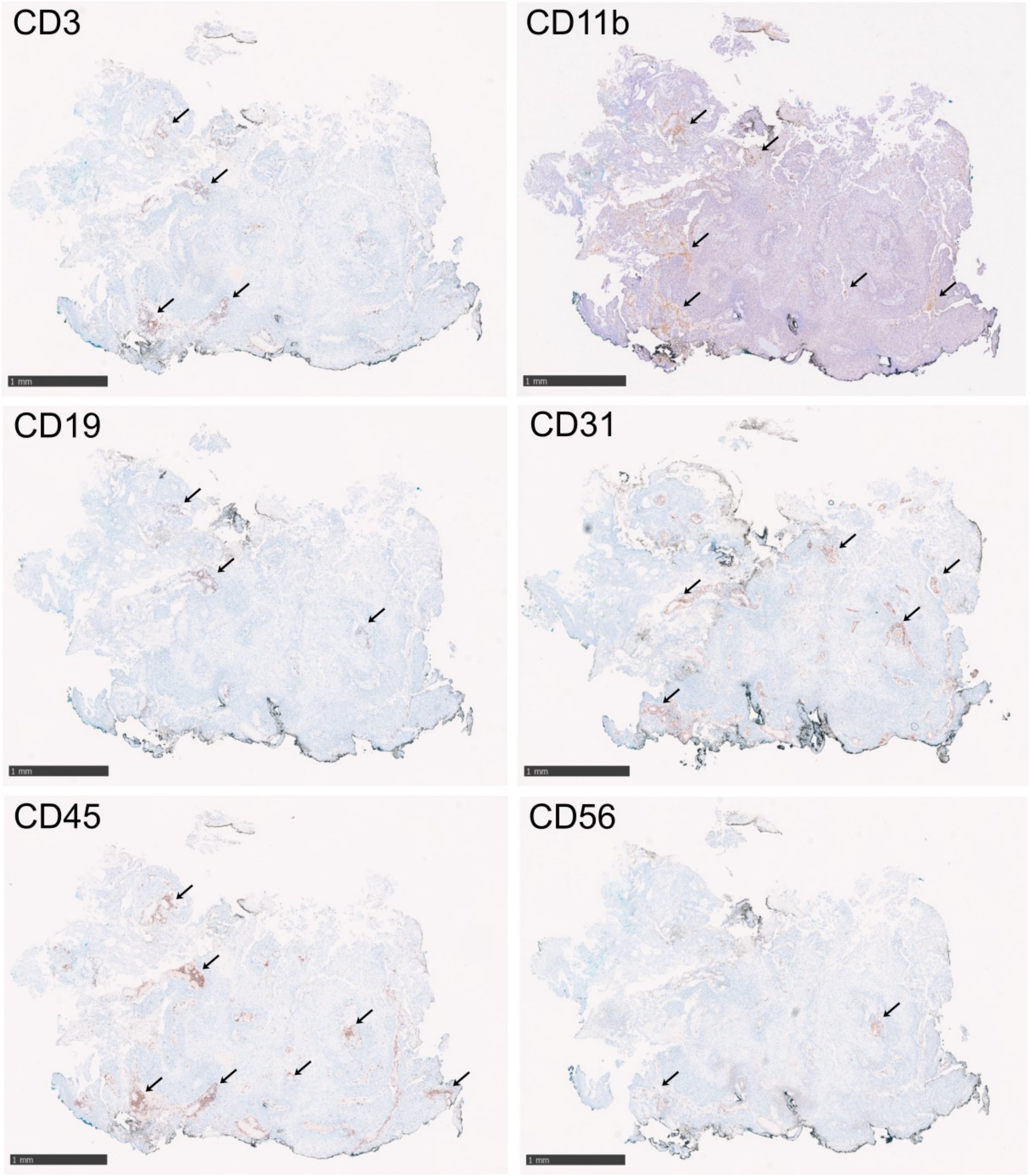

Supplementary Figure 4. Immunohistochemistry of Tumor #2. Immunohistochemistry staining (brown signal) on cervical cancer tissue sections show the expression of multiple markers to identify the presence of various cell types, including CD3 (*e.g.,* T cells), CD11b (*e.g.,* macrophages), CD19 (*e.g.,* B cells), CD31 (*e.g.*, endothelial cells), CD45 (*e.g.,* hematopoietic cells), CD56 (*e.g.,* natural killer cells). Scale bars correspond to 1 mm. Arrows indicate antigen-positive regions.

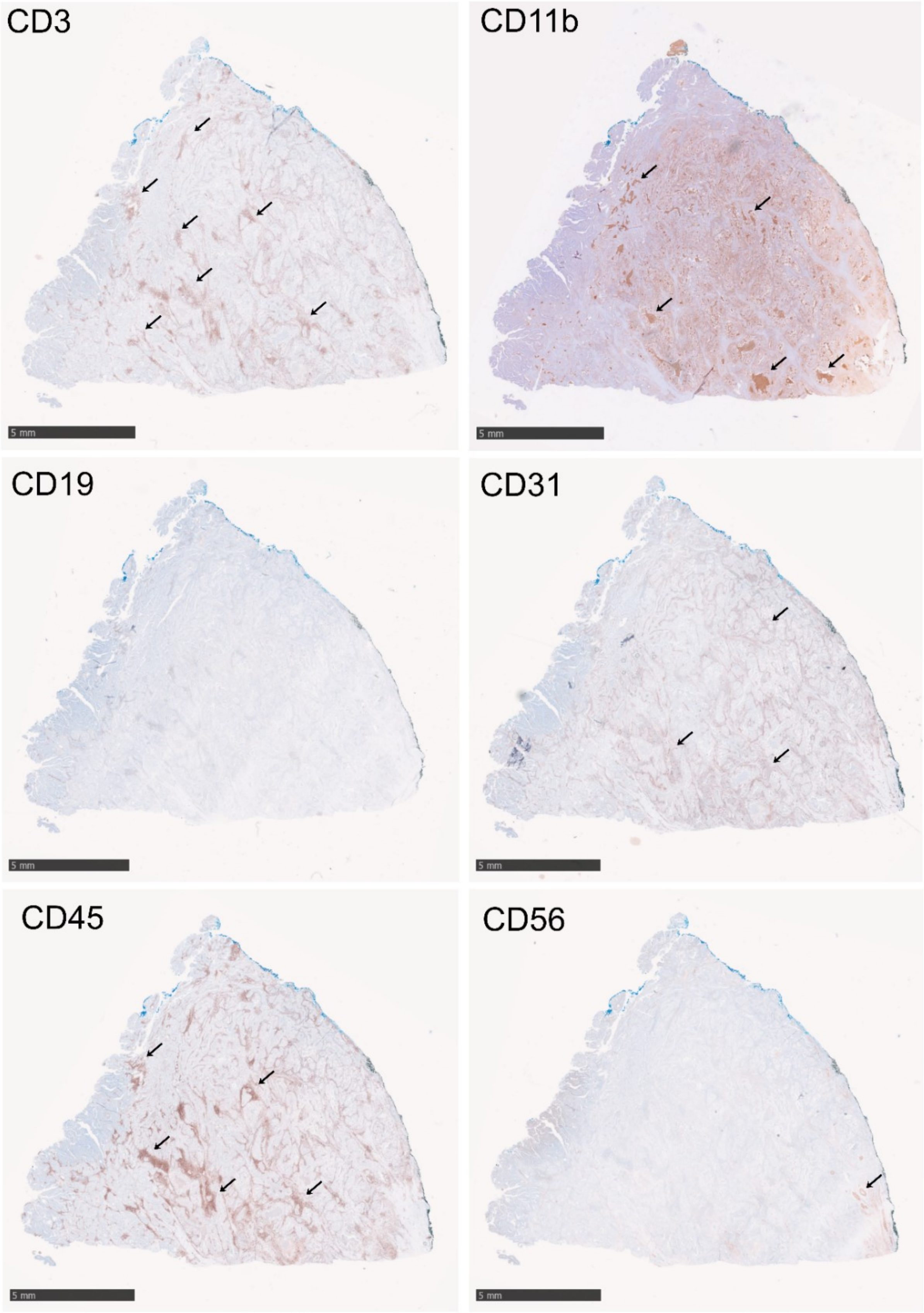
CD3
CD11b
CD19
CD31
CD45
CD56
5 mm

Supplementary Figure 5. Immunohistochemistry of Tumor #3. Immunohistochemistry staining (brown signal) on cervical cancer tissue sections show the expression of multiple markers to identify the presence of various cell types, including CD3 (*e.g.,* T cells), CD11b (*e.g.,* macrophages), CD19 (*e.g.,* B cells), CD31 (*e.g.*, endothelial cells), CD45 (*e.g.,* hematopoietic cells), CD56 (*e.g.,* natural killer cells). Scale bars correspond to 5 mm. Arrows indicate antigen-positive regions.

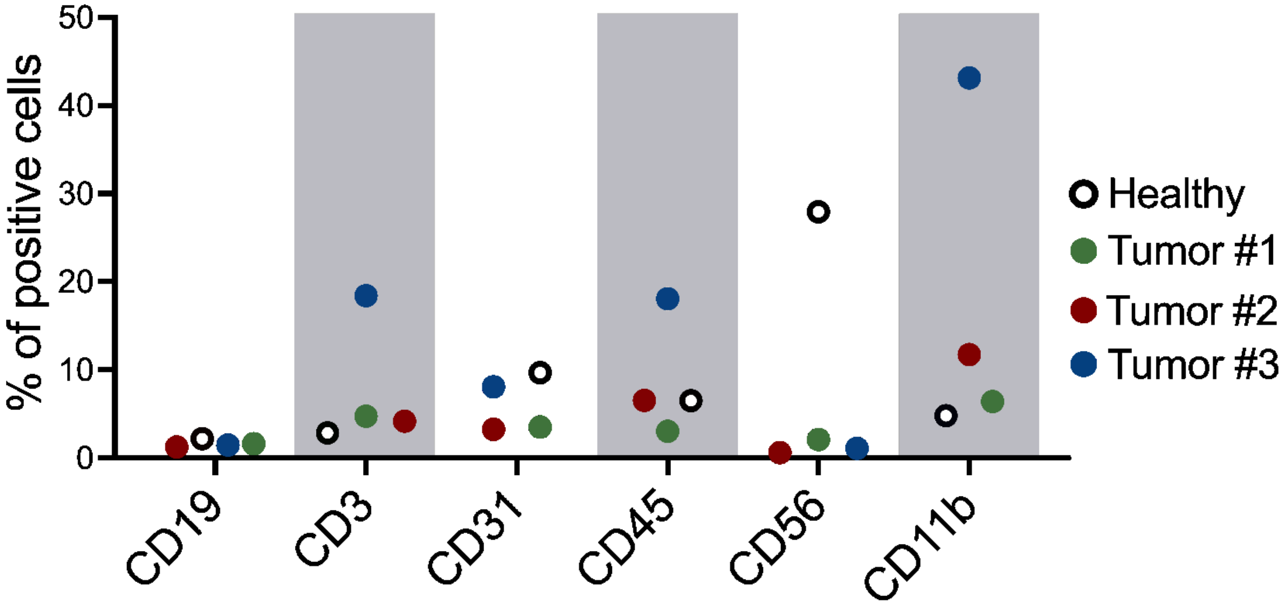

Supplementary Figure 6. Quantification of immune cell markers. Quantitative analysis of immunohistochemistry staining of cervical cancer tissue sections for immune cell markers, including CD3 (*e.g.,* T cells), CD11b (*e.g.,* macrophages), CD19 (*e.g.,* B cells), CD31 (*e.g.*, endothelial cells), CD45 (*e.g.,* hematopoietic cells), CD56 (*e.g.,* natural killer cells, neural cells).

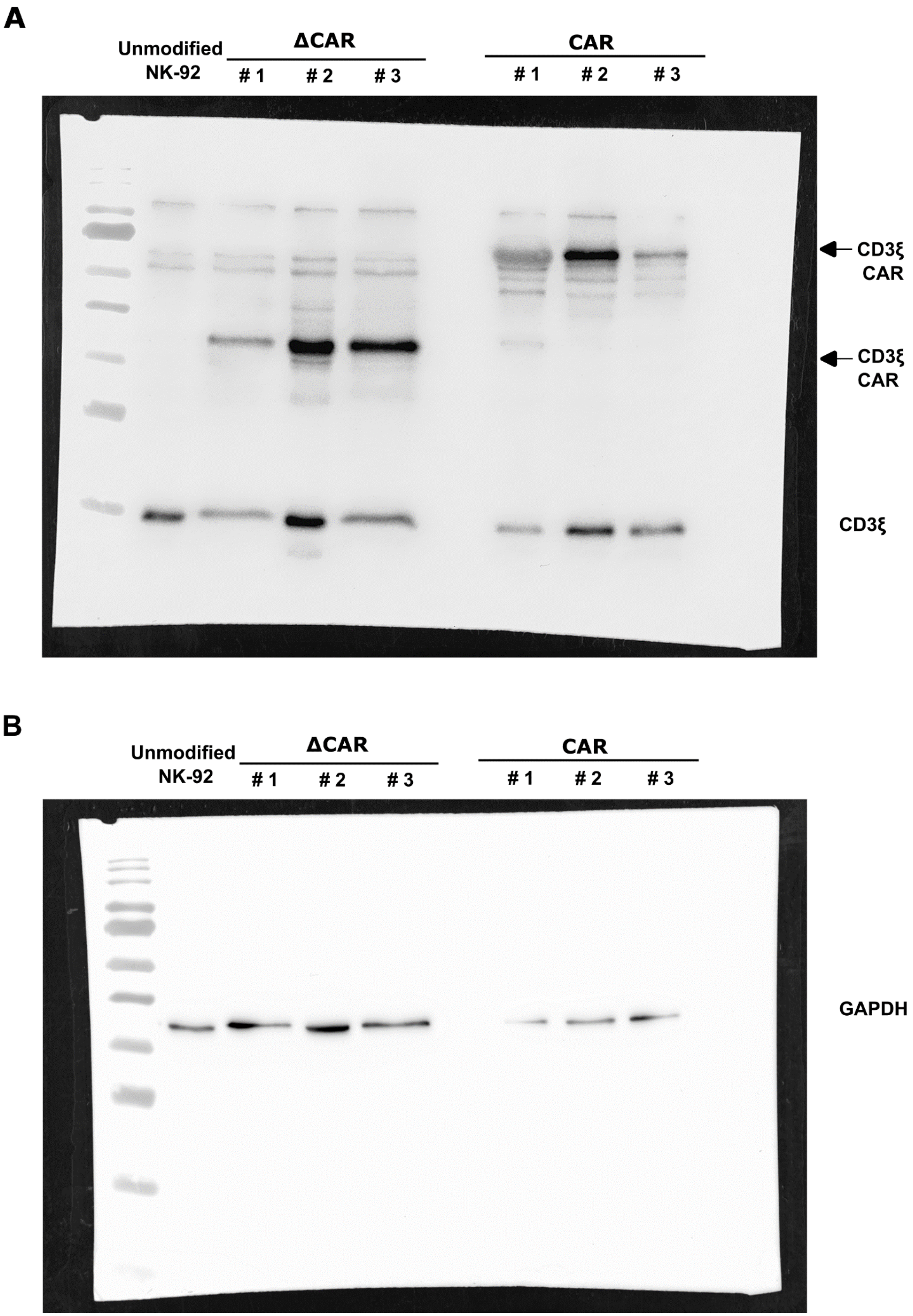

Supplementary Figure 7. Original Western Blot images. Immunoblot before cropping, stained for detection of CD3ζ (**A**) and after stripping stained for loading control GAPDH (**B**). Exposure time 5 seconds.

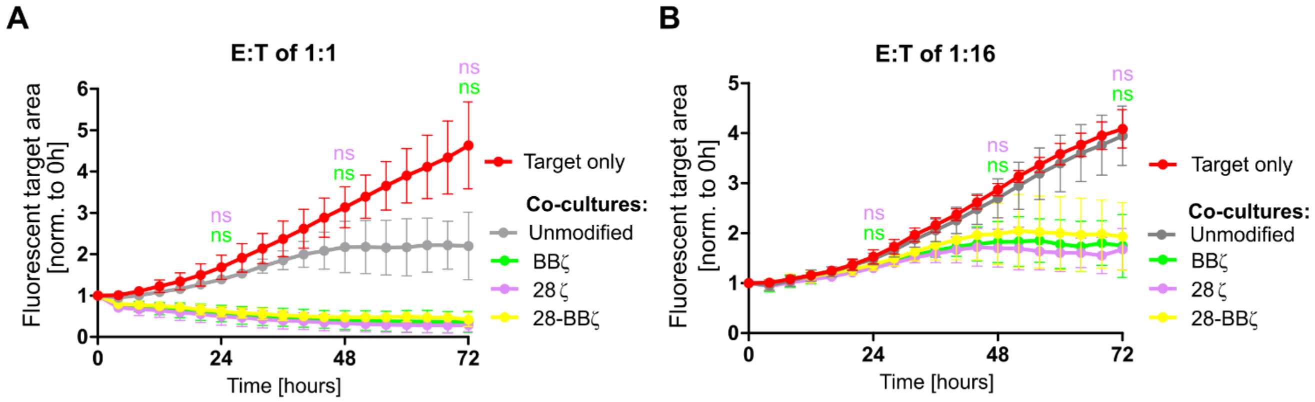

Supplementary Figure 8. Comparison of 2nd and 3rd generation CARs. Live cell imaging-based cytotoxicity assays of anti-Mesothelin CAR-NK-92 cells against mCherry+ SiHa cells. CAR-NK-92 cells were co-cultured with mCherry+ SiHa cells for 72 hours in a 96-well plate at 1:1 (**A**) and 1:16 (**B**) effector to target (E:T) ratios. The anti-tumor activity of the different generations of anti-Mesothelin CAR-NK-92 cells was determined by monitoring the changes in the red-surface area over 72 hours. Data are displayed as mean ± SD, n=3-4, conducted in technical triplicates. Statistical analysis was performed using two-way ANOVA for comparison of 28- BBζ: 3rd generation anti-Mesothelin CAR-NK-92 cells (CD28-41BB-CD3ζ) to BBζ: 2nd generation (41BB-CD3ζ) and 28ζ: 2nd generation (CD28-CD3ζ) anti-Mesothelin CAR-NK-92 cells at 24, 48 and 72 hours. Non-significant differences are indicated as ns.

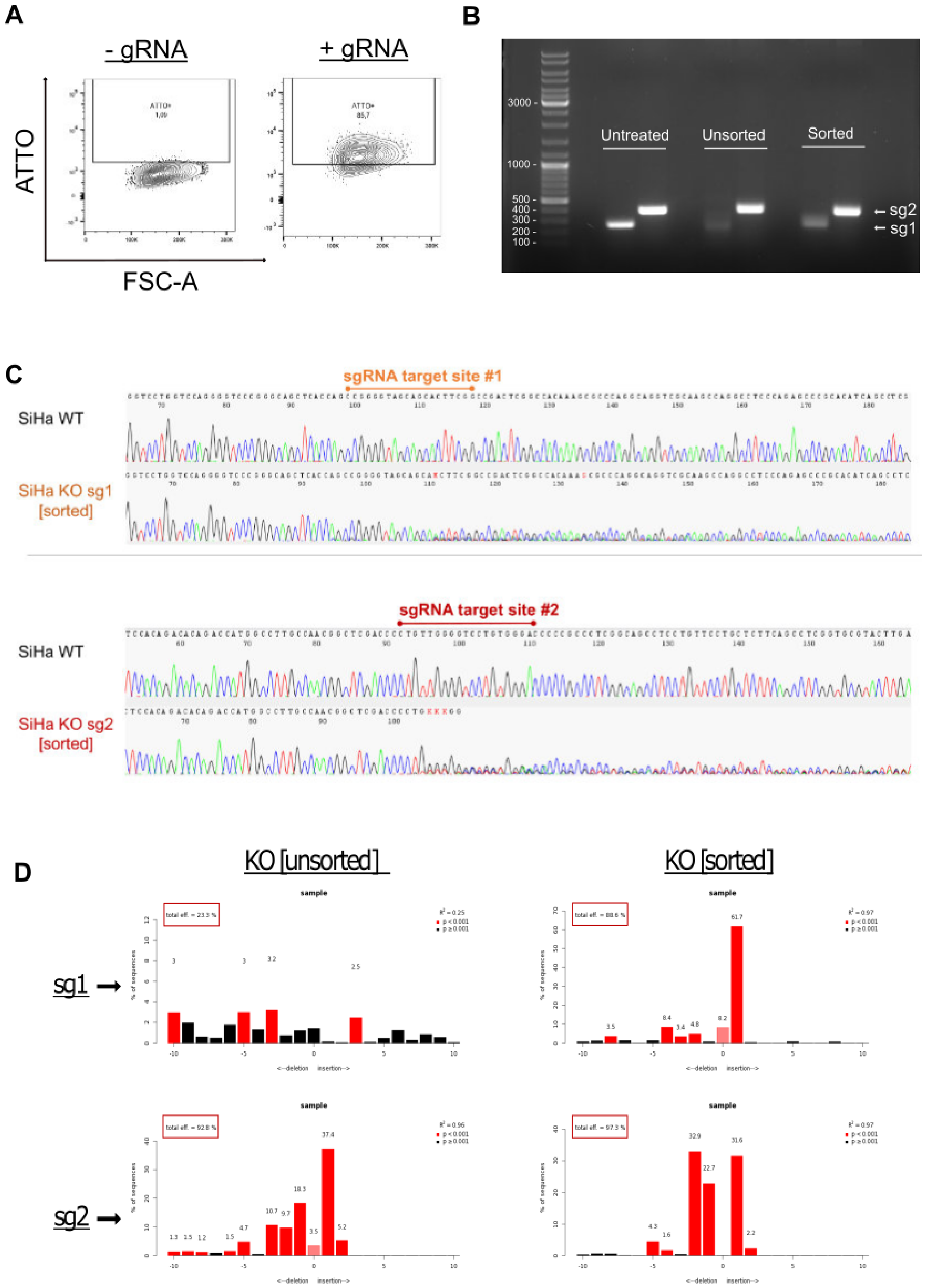
A
- gRNA
+ gRNA
ATTO
FSC-A
ATTO+
1,09
85,7
B
3000
1000
500
400
300
200
100
Untreated
Unsorted
Sorted
← sg2
← sg1
C
sgRNA target site #1
SiHa WT
SiHa KO sg1 [sorted]
sgRNA target site #2
SiHa WT
SiHa KO sg2 [sorted]
D
KO [unsorted]
KO [sorted]
sg1 →
sg2 →
sample
total eff. = 23.3 %
total eff. = 88.6 %
total eff. = 92.8 %
total eff. = 97.3 %
<--deletion insertion-->
% of sequences

Supplementary Figure 9. Knockout of Mesothelin in SiHa cells. Flow cytometric investigation of SiHa cells that were nucleofected with two ATTO-labelled guide RNAs that target different loci of the Mesothelin gene (**A**). Image of a gel showing the amplified DNA, which was used for subsequent sequencing of the gRNA (sg1, sg2) target sites. The DNA was isolated from SiHa cells that were untreated, were nucleofected with the two guide RNAs to knock out Mesothelin but unsorted, and those that were further sorted for cells that do not express Mesothelin as identified by flow cytometry (**B**). Electropherogram showing the CRISPR-Cas9-mediated knockout of Mesothelin on a DNA level. DNA sequences compare wild-type SiHa cells to Mesothelin-KO SiHa cells treated with two different guide RNAs (sg1, sg2) that target different loci (target sites) on the Mesothelin gene (sorted) (**C**). Analysis of the knockout efficiency of unsorted and sorted SiHa cells that were nucleofected with two different guide RNAs (sg1, sg2) using the TIDE program (**D**).

Supplementary Figure 10. Live monitoring of anti-Mesothelin CAR-NK-92 cell cytotoxicity. Videos (4fps) acquired by live-cell imaging capturing the co-cultures of mCherry$^+$ SiHa cells (red) with unmodified NK-92 cells (non-fluorescent) (**A**) or with Mesothelin CAR-NK-92 cells (green) (**B**). Mesothelin-deficient (KO) SiHa cells (red) were stained transiently with a red fluorescent cell tracker and co-cultured with unmodified NK-92 cells (non-fluorescent) (**C**) or with Mesothelin CAR-NK-92 cells (green) (**D**). The cells were co-cultured for 48 hours in a 1:1 E:T ratio; images were acquired using the phase contrast, the red channel (Ex 567-607 nm; Em 622-704 nm), and the green channel (Ex 441-481 nm; Em 503-544 nm) every four hours using the Incucyte® (**A**-**D**). Scale bars correspond to 400 µm. All videos are uploaded individually as MP4 files.

Supplementary Figure 11. Live monitoring of anti-Mesothelin CAR-NK-92 cell cytotoxicity in tumor spheroids. Videos (4fps) acquired by live-cell imaging capturing the co-cultures of spheroids containing mCherry$^+$ Mesothelin$^+$ (red) and BFP$^+$ Mesothelin$^-$ SiHa cells (blue) with unmodified NK-92 (**A**) or with Mesothelin CAR-NK-92 cells (**B**). The cells were co-cultured for 72 hours in a 5:1 E:T ratio; images were acquired using the red (Ex 580-598 nm; Em 612-680 nm) and blue (Ex 370-410 nm, Em 429-462 nm) channels every four hours using the CELLCYTE X™. Scale bars correspond to 100 µm. All videos are uploaded individually as MP4 files.

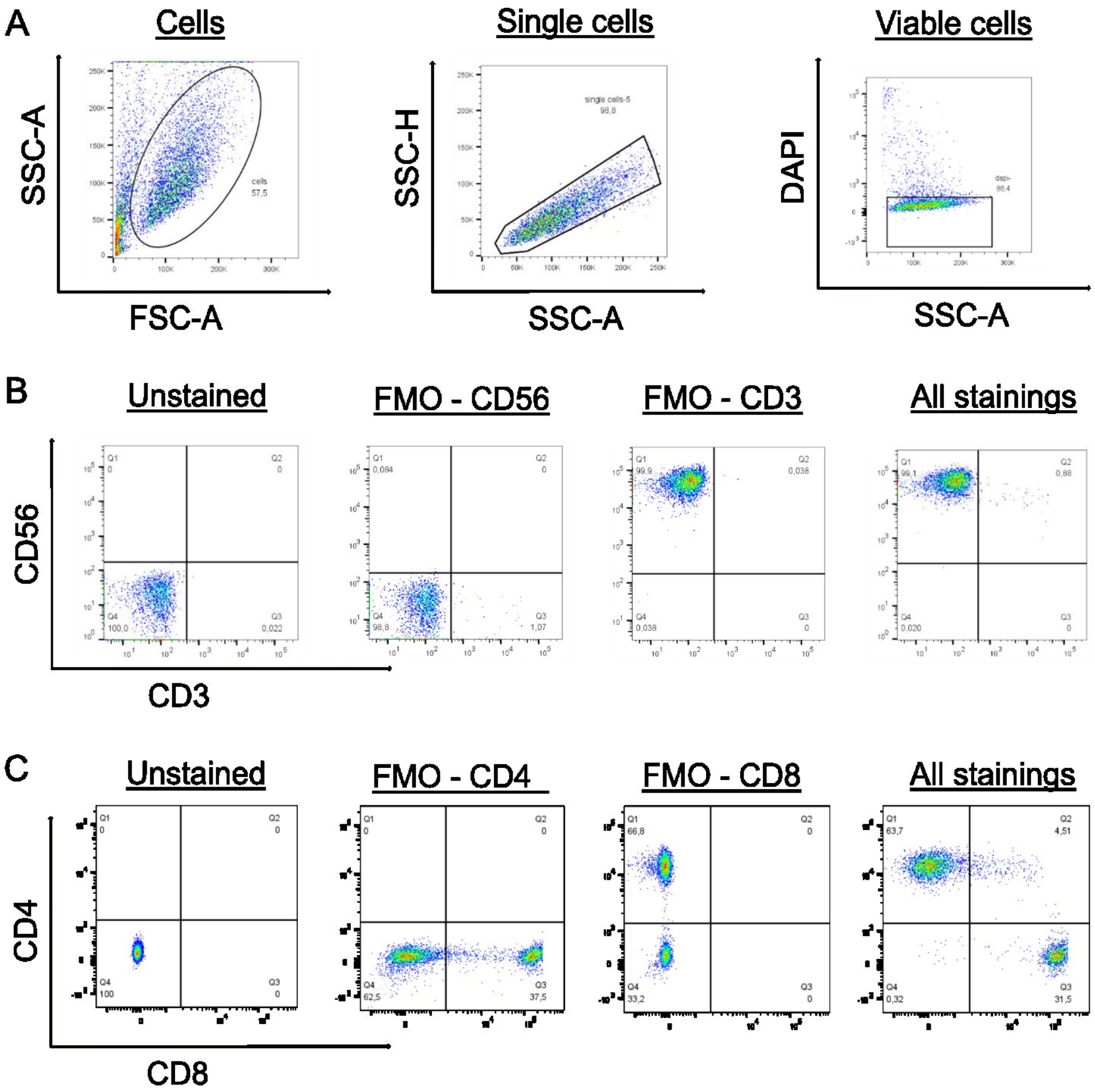

Supplementary Figure 12. Flow cytometric analysis of isolated primary NK and T cells. Evaluation of isolated primary NK and T cells following MACS isolation from cord blood and peripheral blood-derived mononuclear cells. As a gating strategy, cells were distinguished from debris via FSC and SCC parameters. Viable cells were identified by staining negative for DAPI (**A**). CD3 and CD56 expression levels were evaluated to investigate the purity of isolated primary NK cells (**B**). CD4 and CD8 expression levels were evaluated to investigate T cell subtypes (**C**).

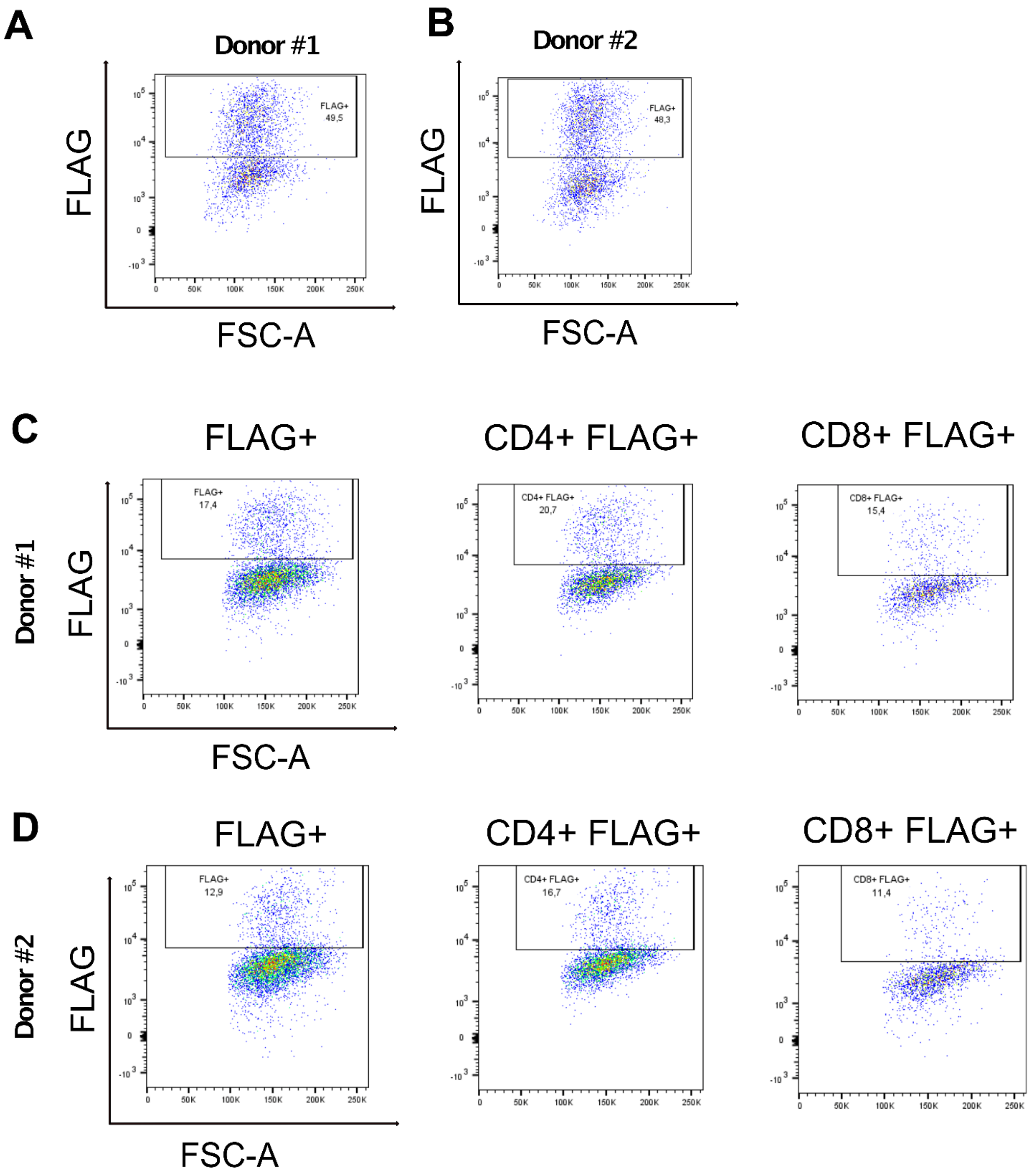

Supplementary Figure 13. Flow cytometric analysis of CAR-transduced primary NK- and T cells. Investigation of peripheral blood-derived primary NK (**A,B**) and T cells (**C,D**) that have been transduced with viral supernatant encoding for anti-Mesothelin CARs. FLAG expression was assessed by flow cytometry for primary NK and T cells derived from two donors. The transduction of $CD4^+$ and $CD8^+$ T cell populations is also displayed (**C,D**).

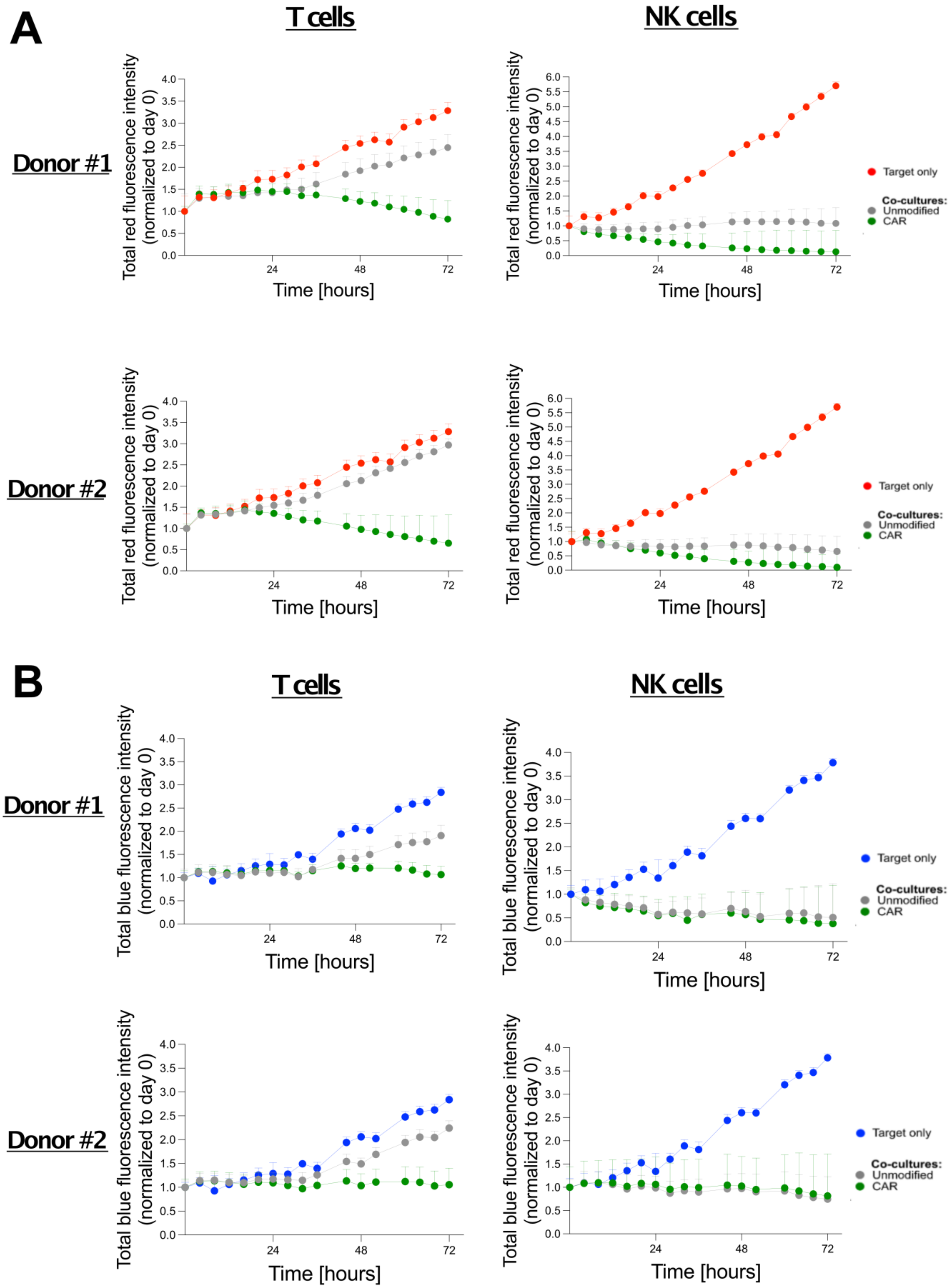
A
T cells
NK cells
Donor #1
Donor #2
Total red fluorescence intensity (normalized to day 0)
Time [hours]
Target only
Co-cultures:
Unmodified
CAR
B
T cells
NK cells
Donor #1
Donor #2
Total blue fluorescence intensity (normalized to day 0)
Time [hours]
Target only
Co-cultures:
Unmodified
CAR

Supplementary Figure 14. Live-cell imaging of primary T and NK cells co-cultured with cervical cancer spheroids. Peripheral blood-derived unmodified and anti-Mesothelin CAR-modified T and NK cells were co-cultured with mCherry^{+} Mesothelin^{+} and BFP^{+} Mesothelin^{-} SiHa cells in a mixed spheroid model. Total red fluorescence intensity (indicative of Mesothelin^{+} SiHa cells) (**A**) and total blue fluorescence intensity (indicative of Mesothelin^{-} SiHa cells) (**B**) were quantified and normalized to day 0 using the CELLCYTE Studio software. Data are displayed as mean ± SD. Each graph depicts the results from an independent pPB-T and pPB-NK donors accomplished in four technical replicates, n=1.
